# Parent-mediated social communication therapy for young children with autism (PACT): long-term follow-up of a randomised controlled trial

**DOI:** 10.1016/S0140-6736(16)31229-6

**Published:** 2016-11-19

**Authors:** Andrew Pickles, Ann Le Couteur, Kathy Leadbitter, Erica Salomone, Rachel Cole-Fletcher, Hannah Tobin, Isobel Gammer, Jessica Lowry, George Vamvakas, Sarah Byford, Catherine Aldred, Vicky Slonims, Helen McConachie, Patricia Howlin, Jeremy R Parr, Tony Charman, Jonathan Green

**Affiliations:** a Institute of Psychiatry, Psychology and Neuroscience, Kings College London, UK; b Newcastle University, Newcastle, UK; c Northumberland Tyne and Wear NHS Trust, Newcastle upon Tyne, UK; d University of Manchester, Manchester, UK; e Evelina London Children's Hospital, Guys and St Thomas University NHS Trust, London, UK; f Royal Manchester Children's Hospital and Manchester Academic Health Sciences Centre, Manchester, UK

## Abstract

**Background:**

It is not known whether early intervention can improve long-term autism symptom outcomes. We aimed to follow-up the Preschool Autism Communication Trial (PACT), to investigate whether the PACT intervention had a long-term effect on autism symptoms and continued effects on parent and child social interaction.

**Methods:**

PACT was a randomised controlled trial of a parent-mediated social communication intervention for children aged 2–4 years with core autism. Follow-up ascertainment was done at three specialised clinical services centres in the UK (London, Manchester, and Newcastle) at a median of 5·75 years (IQR 5·42–5·92) from the original trial endpoint. The main blinded outcomes were the comparative severity score (CSS) from the Autism Diagnostic Observation Schedule (ADOS), the Dyadic Communication Assessment Measure (DCMA) of the proportion of child initiatiations when interacting with the parent, and an expressive-receptive language composite. All analyses followed the intention-to-treat principle. PACT is registered with the ISRCTN registry, number ISRCTN58133827.

**Findings:**

121 (80%) of the 152 trial participants (59 [77%] of 77 assigned to PACT intervention *vs* 62 [83%] of 75 assigned to treatment as usual) were traced and consented to be assessed between July, 2013, and September, 2014. Mean age at follow-up was 10·5 years (SD 0·8). Group difference in favour of the PACT intervention based on ADOS CSS of log-odds effect size (ES) was 0·64 (95% CI 0·07 to 1·20) at treatment endpoint and ES 0·70 (95% CI −0·05 to 1·47) at follow-up, giving an overall reduction in symptom severity over the course of the whole trial and follow-up period (ES 0·55, 95% CI 0·14 to 0·91, p=0·004). Group difference in DCMA child initiations at follow-up showed a Cohen's *d* ES of 0·29 (95% CI −0.02 to 0.57) and was significant over the course of the study (ES 0·33, 95% CI 0·11 to 0·57, p=0·004). There were no group differences in the language composite at follow-up (ES 0·15, 95% CI −0·23 to 0·53).

**Interpretation:**

The results are the first to show long-term symptom reduction after a randomised controlled trial of early intervention in autism spectrum disorder. They support the clinical value of the PACT intervention and have implications for developmental theory.

**Funding:**

Medical Research Council.

## Introduction

Autism spectrum disorder is a common neurodevelopmental disorder that affects about 1% of children and young people.^1^, ^2^ The natural history of the disorder is usually enduring and has serious effects on development; lifetime costs (including health, education, social care, family out-of-pocket expenses and productivity losses) are estimated to be between GB£1 million and £1·5 million in the UK and between US$1·4 million and $2·4 million in the USA.^3^ Effective early treatment that alters the long-term course of the disorder would therefore have great potential benefits for individuals, families, and society, but has been difficult to demonstrate. Evidence shows that a range of psychosocial intervention approaches can have short-term effects on various developmental indicators that are thought to be important for later autism outcomes, such as parent-child joint engagement, social communication, child symbolic play, and social imitation. Follow-up data from one study showed improved language outcomes 5 years after the initial treatment endpoint.^4^ However, evidence is scarce as to whether such intermediate effects are associated with reduced autism symptom severity or improved longer-term post-treatment symptom outcomes.

In a Cochrane review,^5^ Oono and colleagues identified six, mainly small, studies of parent-mediated interventions that addressed autism severity as a treatment outcome according to various blinded child assessment measures and non-blinded parent-reported measures. A random-effects meta-analysis of the reported mean symptom scores suggested an overall effect of intervention compared with control in terms of reducing symptom severity (standard mean difference −0·30, 95% CI −0·52 to −0·08, p<0·05; combined n=316). Three of these studies used blinded symptom outcome measures; these were the pilot study^6^ and subsequent larger trial^7^ of a 12 month pre-school autism communication intervention (Preschool Autism Communication Trial; PACT). In a separate meta-analysis by the National Institute for Health and Care Excellence (NICE),^8^ the weighted combined effect size of the treatment used in these PACT studies was −0·29 (95% CI −0·59 to 0·00, p=0·05; n=180) in favour of the intervention, as assessed with a standard blinded measure of the social communication-specific symptoms of autism (Autism Diagnostic Observation Schedule [ADOS]-Generic [ADOS-G]).^9^ A third study also used the ADOS measure, and the results showed no effect on the symptom endpoint after 2 years of intensive treatment with the Early Start Denver Model (ESDM) intervention (n=48).^10^ This trial of ESDM was the basis for one of two longer-term follow-up studies of autism symptoms to have been attempted so far. ADOS assessment was done at a mean age of 6 years, 2 years after the end of the intervention.^11^ In individuals who were followed up, the investigators reported significantly greater symptom reduction in intervention participants (n=21) than in regular care participants (n=18), although inferences from these findings were limited by the small sample size and lack of intention-to-treat analysis. In the other follow-up study of autism symptoms,^12^ no group differences were found 12 months after a 12 week teacher-mediated intervention (n=33) compared with controls (n=27) in terms of non-blinded parent-rated or teacher-rated autism symptoms assessed with the Social Communication Questionnaire at age 5 years. Other reports of long-term follow-up after intensive behavioural programmes have not addressed symptoms, but have suggested ongoing effects on IQ and adaptive behaviour,^13^ albeit on the basis of non-experimental studies^14^ or very small samples.^15^

**Research in context**

**Evidence before this study**

There has been relatively little study of long-term symptom outcomes after autism treatment. A Cochrane review from 2013 investigated parent-mediated intervention for young children with autism spectrum disorder and included six randomised controlled trials that reported autism symptom outcomes. Overall, the review showed reduced symptom severity with intervention compared with control conditions (standard mean difference −0·30, 95% CI −0·52 to −0·08, p<0·05; combined n=316) on random-effects meta-analysis. Additionally we searched for intervention trials reporting on longer-term symptom follow-up after parent-mediated, teacher-mediated, or therapist-mediated early interventions for young children with autism spectrum disorder. We searched PubMed, Web of Science, PsycINFO, and MEDLINE for articles published between Jan 1, 2000, and May 6, 2016, using the search terms “autism”, “early”, “intervention”, “long-term”, and “outcomes” and selecting studies that reported autism severity. We found two studies, one of which reported greater symptom reduction (assessed in a blinded manner) in participants followed up from the original intervention (n=21) compared with regular care (n=18) groups at a mean age of 6 years, two years after a 2 year intensive pre-school intervention. The other study found no effect on parent-reported or teacher-reported symptom questionnaires at 12 months after a 12 week preschool intervention (n=33) versus control (n=27).

**Added value of this study**

The two previous follow-up studies to test the longer-term effects on symptoms after interventions for autism spectrum disorder in young children were marked by small sample size and relatively short follow-up periods. Our study contributes now to the scientific literature in terms of its sample size (n=152) and the longer-term nature of the follow-up (nearly 6 years from treatment endpoint, with mean age of 10 years at follow-up) with blinded symptom ascertainment.

**Implications of all the available evidence**

Previous evidence from trials has suggested that early intervention can result in short-term symptom reduction in young children with autism spectrum disorder. We now show that a 12 month parent-mediated preschool intervention can produce sustained improvement in child autism symptoms and social communication with parents, which remained at nearly 6 years after the end of treatment. These findings support the potential long-term effects and value of early parent-mediated interventions for autism.

In this context, the aim of our present study was to investigate the long-term outcomes of the largest of the trials done so far, PACT, which assessed the effects of a developmentally targeted social communication intervention on autism symptoms and other outcomes in children aged 2–4 years. The video-aided intervention in PACT works with the parent rather than directly with the child, aiming to optimise developmentally relevant parent interactive behaviours, which will in theory enhance parent–child dyadic interaction, consequently improving child communication and more general autism symptoms. The logic of this treatment procedure is that the dyadic interaction is the proximal target of the intervention and the delivery mechanism by which the child gains benefit. The initial PACT trial tested this intervention against treatment as usual at three sites in a randomised controlled trial with two parallel arms (n=152).^6^ Trial data and associated mediation analysis^16^ supported this model of the hypothesised treatment mechanism. A strong effect on the targeted parental behaviour (parental synchronous response to child communication during interaction) mediated 71% of improvement in child communication with the parent during interaction. In turn, this improvement in child dyadic communication mediated 73% of the independently assessed symptom change in the child in a different context.

Our predefined hypotheses were that, at follow-up, we would find enhanced effects of the intervention on the autism symptom outcome; continuation of the initial intervention effects on dyadic communication (parent synchrony, child communication initiations) and enhanced effects on reported adaptive functioning; and sustained reduction in restricted and repetitive behaviours of the child, accompanied by reduced anxiety, which has been associated with restricted and repetitive behaviours in some studies.^17^ The design of the present long-term follow-up of the PACT trial therefore combined analysis based on the initial randomised intervention allocation with prospective repeated measure follow-up to test for downstream developmental effects of the intervention on later autism symptoms.

## Method

### Study design and participants

PACT was a randomised controlled trial done at three specialist centres in the UK (London, Manchester, and Newcastle).^6^ The PACT trial and follow-up study were approved by the Central Manchester Multicentre Research Ethics Committee (Manchester, UK). Written consent to participate was provided by at least one parent in each family enrolled in the study. The protocol for this study is available in the appendix.

Children aged from 2 years to 4 years and 11 months were recruited if they met the criteria for so-called core autism in accordance with the international standard diagnostic tests (social and communication domains of the ADOS-G,^9^ and two of three domains of the Autism Diagnostic Interview Revised [ADI-R] algorithm).^18^ Exclusion criteria were children with a twin with autism, non-verbal age equivalent to 12 months or younger (Mullen scales), epilepsy requiring medication, severe sensory impairment, or severe mental illness in a parent. Participating parents spoke English with their child. For this follow-up study, we attempted to trace all participants of the original trial unless they had previously withdrawn consent for follow-up.

Full details of the trial design, PACT intervention protocol and the between-group balance in nature and hours of treatment as usual received have been published previously.^6^ Assessment of primary outcomes was done by assessors unaware of treatment allocation.

### Procedures

The PACT intervention is a 1 year developmentally focused social communication intervention programme for young children that consists of 12 therapy sessions (each 2 h long) over 6 months, followed by monthly support and extension sessions for a further 6 months, as described previously.^6^ Additionally, the parents agree to do 20–30 min per day of planned practice activities with the child. The assessments at follow-up were done by trained assessors in the centres, with occasional home visits if needed by families.

### Outcomes

The primary outcomes were autism symptom severity, assessed with the ADOS Comparative Severity Score (CSS),^19^, ^20^ parent-child dyadic communication, using the Dyadic Communication Measure for Autism (DCMA), and language composite scores; calculated at baseline, the original study endpoint, and follow-up. The updated ADOS CSS coding, which has been published since our original report, reflects current Diagnostic and Statistical Manual of Mental Disorders, 5th Edition (DSM-5)-defined autism spectrum disorder criteria by combining social communication and restricted and repetitive behaviours (which were reported separately in the original PACT endpoint report)^6^ into an overall symptom severity score. This system allows for comparison across different developmentally staged ADOS modules. Scores range from 1 to 10, where scores of 1 and 2 represent minimal-to-no evidence of autism; 3 and 4 represent low severity; 5–7 represent moderate severity; and 8–10 represent high severity. Assessor training achieved the required reliability as shown by intraclass correlations from 52 double-ratings of 12 children assessed during the main trial and 50 ratings of 25 children assessed at follow-up of 0·73 (95% CI 0·58 to 0·84). The severity scales can also be calculated for the two component symptom domains; social-affect (SA) and restricted and repetitive behaviours (RRB).^9^

Parent–child dyadic communication was assessed via measurement of an 8-min video sample of carer–child naturalistic play, using the DCMA.^6^, ^21^ This measurement was obtained in a context independent of the context of therapy, with separate protocol and play materials to avoid circularity with the therapy process itself. Research assessors were blind to treatment allocation. Interactions were coded as child communication initiation with the parent, calculated as a proportion of all child acts, logit-transformed for normality; parental synchronous response with child, calculated as the logit-transformed proportion of all parent acts; and conversational turns (follow-up only) between parent and child, as a log-transformed number of turns during a session. Child and parent variables are defined independently from each other. Assessor training achieved the required reliability as shown by intraclass-correlations from 22 double-ratings of 0·80 (95% CI 0·63 to 0·93), 0·76 (0·61 to 0·91), and 0·90 (0·63 to 0·98) respectively.

Child language was assessed with a composite from six indicators; four subscales of the Child Evaluation of Language Fundamentals (CELF-4) tests^22^ and expressive and receptive language raw scores from the one-word test.^23^

Vineland Adaptive Behavior Composite (ABC) standard score^24^ (child adaptive function) was a secondary outcome, assessed by a teacher blinded to allocation.

Other secondary outcomes were assessed by parents in a non-blinded manner. Autism symptoms were assessed by the parent with the Social Communication Questionnaire (SCQ).^25^ Restricted and repetitive behaviours, which consist of sensory and motor behaviours and insistence on sameness or circumscribed interests, were scored with the Repetitive Behaviour Questionnaire (RBQ).^26^ Social difficulties were assessed with the pro-social and peer problems five-item subscales of the Strength and Difficulties Questionnaire (SDQ),^27^ and adaptive behaviour outcomes were assessed with parent-rated Vineland ABC standard score.^24^ Comorbid psychopathology was recorded as bands defined by the Development and Well-Being Assessment (DAWBA)^28^ indicating the risk of four common co-occurring disorders (depression, conduct or oppositional disorders, hyperkinesis, and anxiety or obsessive compulsive disorder [OCD]) or groups of disorders as classified by the International Statistical Classification of Diseases and Related Health Problems 10th Revision (ICD-10). Data for parent-reported outcomes were collected by interview, online, and post via stamped-addressed envelopes.

### Statistical analysis

Departures from the analysis plan are declared in the Methods section and in the statistical analysis plan in the appendix. We present descriptive statistics for participants with data at follow-up. We used logistic regression to identify baseline characteristics associated with dropout and measures for which treatment groups were not balanced at follow-up. Analysis of all outcomes followed intention-to-treat principles. We estimated differences between the intervention groups at the mean follow-up age for the whole sample, under the assumption of attrition being at random, by the use of the maximum-likelihood method, adjusting for centre and variables for which groups were not balanced at endpoint or follow-up. We checked these estimates against whole-sample analyses in which missing observations were completed by multiple imputation using chained equations (*mi* command in Stata). Where available, we included repeated measures in mixed-effects models to improve the efficiency of estimation of group differences at follow-up, to provide additional effect estimates at earlier time points, to increase the likely validity of the missing-at-random assumption, and to allow calculation of the area-under-the-curve (AUC) treatment effect estimates. We modelled the effects of time by piecewise slopes between each assessment for each treatment group, with the AUC calculated as the sum of the triangular or trapezoidal areas between the groups. These AUC effect estimates provide a principled basis for an overall mean effect for unequally spaced measures that summarise treatment effect over the whole trial from baseline to follow-up. AUC effect estimates were obtained by use of the *lincom* post-estimation command.

Of the blinded outcomes, we analysed the ADOS CSS with mixed-effect ordinal logistic regression (*xtologit*) with random intercept and robust standard errors. To make the subject-specific model log-odds ratio estimates more interpretable, we report the coefficients after the rescaling to the smaller approximate marginal (population-average) log-odds ratios.^29^ The treatment effect proportional-odds assumption was checked.^30^ Planned secondary analysis tested the contribution of social communication and repetitive behaviour symptom domains to the overall effect.

We normalised the DCMA interaction measures with an empirical logit transformation and analysed them with mixed-effects regression with random intercept (*xtmixed*). We used a structural equation model (described in the appendix) to estimate the group difference in the mean of a factor defined by each of the six language scores (*sem*). Of the non-blinded outcomes, the parent Vineland ABC scores were also analysed with a mixed-effects model. Because they lacked baseline measurement, we analysed the teacher Vineland ABC, SCQ, and RBQ scores by simple regression, and the DAWBA risk bands and SDQ peer problems and prosocial subscales by ordinal logistic regression. Cohen's *d* effect size estimates used the pooled baseline standard deviation, or if unavailable the standard deviation at outcome conditional on treatment. All confidence intervals are 2·5% and 97·5% percentiles from 1000 bootstrap samples (with replacement).

Statistical analyses were done by AP and GV using Stata version 13. Further details of the statistical analyses are available in the appendix. PACT is registered with the ISRCTN registry, number ISRCTN58133827.

### Role of the funding source

The funder of the study had no role in study design, data collection, data analysis, data interpretation, or writing of the report. AP and JG had full access to all the data in the study and had final responsibility for the decision to submit for publication.

## Results

**Figure 1:**
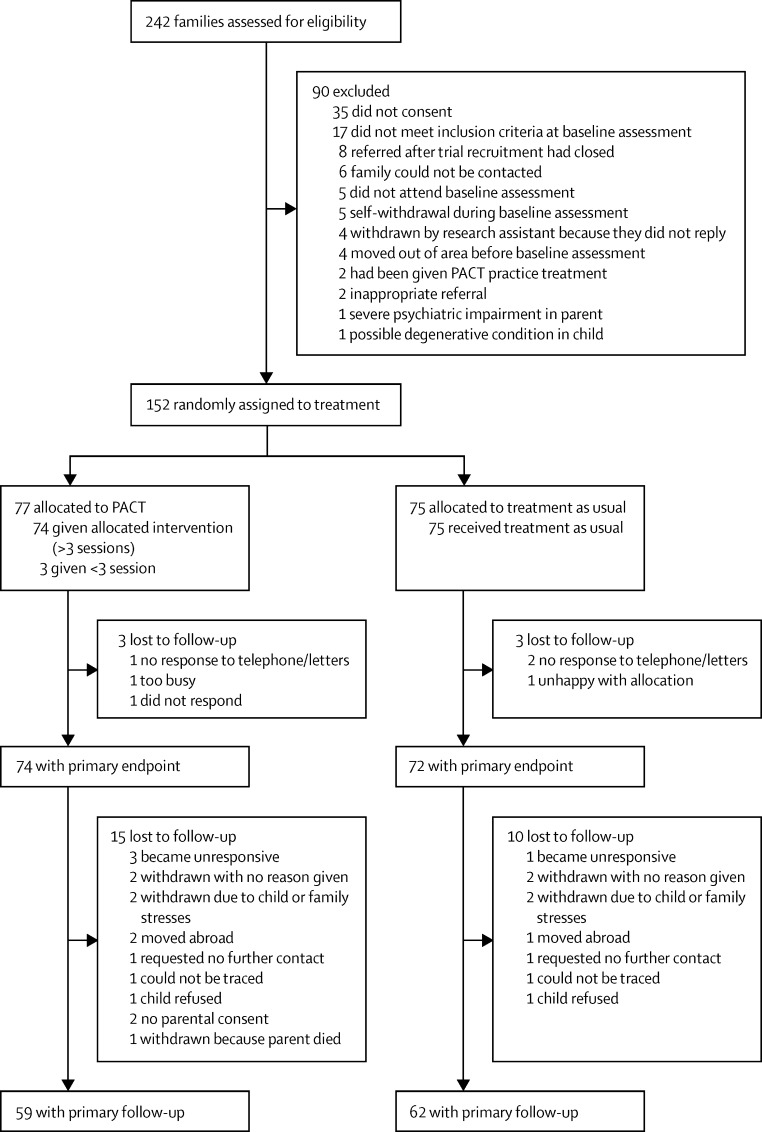
Profile of PACT trial plus follow-up study

152 participants were initially recruited into PACT between September, 2006, and February, 2008. 77 participants were assigned to the PACT intervention and 75 were assigned to treatment as usual. Figure 1 shows the flow of participants through the trial and follow-up. We were able to trace 144 (95%) of 152 participants, 126 (88%) of whom consented to participate in the current study. Of those randomly assigned, the follow-up ADOS assessment was completed for 59 (77%) of 77 participants assigned to the PACT intervention group and 62 (83%) of 75 participants in the treatment as usual group. At follow-up, 43 participants were assessed with module 1 of ADOS, 22 were assessed with module 2, and 56 were assessed with module 3. The median length of follow-up (baseline to follow-up) was 82 months (IQR 78–85), with median time from treatment endpoint to follow-up of 69 months (65–71). The mean age of participants at follow-up was 10·5 years (SD 0·8). Table 1 shows descriptive statistics by intervention group at baseline (all complete) and follow-up. The 31 participants lost to follow-up (six during the trial and 25 during follow-up) had no significant associations with treatment group, centre, autism severity, level of adaptive functioning, or any of the demographic measures shown in table 1. In individuals with follow-up data, participants in the PACT intervention group were more likely to be boys (p=0·05), from two-parent households (p=0·02), and to have parents with higher education (p=0·001) at baseline.

**Figure 2:**
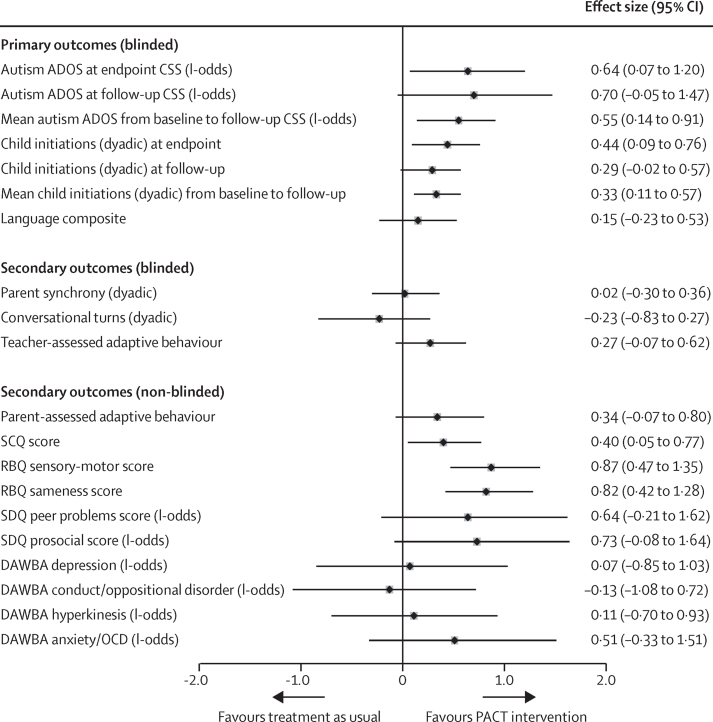
Effect estimates and 95% bootstrap CIs Plot shows log-odds and Cohen's *d* effect sizes for PACT versus treatment as usual comparisons (effect estimates and 95% bootstrap CIs). PACT=preschool autism communication trial. ADOS CSS=Autism Diagnostic Observation Schedule Comparative Severity Score. SCQ=Social Communication Questionnaire. RBQ=Repetitive Behaviour Questionnaire. SDQ=Strength and Difficulties Questionnaire. DAWBA=Development and Well-Being Assessment. OCD=obsessive compulsive disorder.

**Figure 3:**
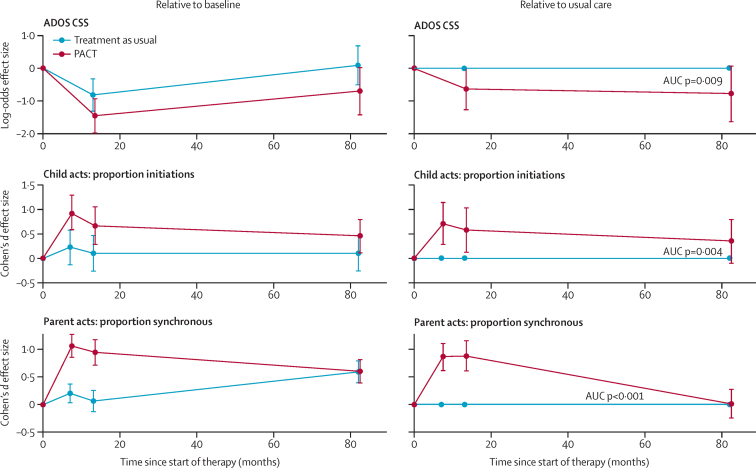
Course of outcomes by group from baseline to follow-up Group time-paths relative to baseline (left) and PACT relative to treatment as usual (right). Bars represent time-specific estimates with 95% CIs from repeated measures models and p values for area test of no-difference between group profiles. PACT=preschool autism communication trial. ADOS CSS=Autism Diagnostic Observation Schedule Comparative Severity Score. AUC=area-under-curve estimation.

Table 2 shows descriptive data by treatment group for the follow-up outcomes. Figure 2 shows effect estimates (both log-odds ratio and Cohen's *d*) after adjustment for the measures in table 1 that were unbalanced. Very similar estimates were obtained with multiple imputed outcomes (appendix). The descriptive statistics (table 2) and effect estimates (figure 2) show that, at the original treatment endpoint, the intention-to-treat estimate of autism symptom severity measured with ADOS CSS showed a larger reduction (marginal log-odds effect size [ES] 0·64, 95% CI 0·07 to 1·20) than that previously reported with the social communication algorithm score alone (Cohen's *d* ES of 0·24, 95% CI −0·59 to 0·11, in favour of PACT),^6^ with no evidence of effects varying with severity (proportional-odds test p=0·23). This estimate corresponded to an endpoint difference in the proportion of high severity CSS symptom scores in each group (44% in the treatment as usual group and 29% in the PACT intervention group) and represented a difference in favour of the PACT intervention of 15·4% (95% bootstrap CI 1·2 to 29·7) for intervention versus treatment as usual. At follow-up, the point estimate of ADOS CSS showed a continuing group difference in symptoms, but with a CI that included the null (marginal log-odds ES 0·70, 95% CI −0·05 to 1·47). Again, there was no evidence of effects varying with severity (proportional-odds test p=0·154). The proportion of high severity symptom scores in each group had increased (63% in the treatment as usual group and 46% in the PACT intervention group); a group difference of 17·2% (95% CI −2·9 to 37·3) in favour of intervention versus treatment as usual. Figure 3 shows the course of autism symptoms by group from baseline to trial endpoint, and then to follow-up. The combined mean treatment effects on symptom severity, as estimated from the AUC, showed significantly lower severity scores in the PACT intervention group compared with the treatment as usual group (marginal log-odds ES 0·55, 95% CI 0·14 to 0·91; p=0·009). Secondary analysis (appendix) showed that changes in the social communication and restricted and repetitive behaviour symptom domains had contributed to this overall effect.

Figure 3 also shows the pattern for child dyadic communication initiation. The group difference at follow-up (Cohen's *d* ES 0·29, 95% CI −0·02 to 0·57) was smaller than at endpoint (ES 0·44, 95% CI 0·09 to 0·76), but the mean treatment effect over the whole trial and follow-up was clear (ES 0·33, 95% CI 0·1 to 0·6, p=0·004).

At follow-up we found no trace of the group difference in parent synchrony that was previously reported^6^ at the trial endpoint (table 2, figure 2). However, when the overall time period is taken into account (figure 3), the effects of intervention were significant (ES 0·61, 95% CI 0·38 to 0·86, p<0·0001). The point estimate of effect on teacher-rated adaptive behaviour at follow-up showed an ES of 0·27 (95% CI −0·07 to 0·62) for PACT treatment versus treatment as usual. Correlations among all six language indicators (see methods) exceeded 0·7 (Cronbach α=0·88) and gave an exploratory factor analysis with a dominant first factor (eigenvalue 4·82; appendix). This child language outcome composite showed no evidence of group difference (ES 0·15, 95% CI −0·23 to 0·53).

Non-blinded parent-ratings of autism symptoms (SCQ and RBQ; table 2, figure 2) showed clear and substantial treatment effects at follow-up. Parent report of adaptive behaviour, peer problems and prosocial behaviour showed moderate to substantial point estimate trends in favour of treatment, but CI's on all of these results included null or small negative effects. There was no evidence of treatment effect on co-occurring mental health problems on DAWBA.

## Discussion

We sought to assess the long-term outcomes of the preschool PACT intervention on autism symptoms and other outcomes. The results of our investigation show a treatment effect to reduce autism symptom severity at treatment endpoint, which remained almost 6 years later, giving a clear averaged treatment effect over the total period. The effect was apparent across both autism social-communication and repetitive symptom domains. A similar treatment effect is also seen in parent-reported symptom measures at follow-up which, although unblinded, have the potential complementary strengths of being service-user outcome measures and being based on knowledge of the child in naturalistic settings.

To our knowledge, this study is the first study to report long-term symptom outcomes to middle childhood (7–11 years) following a randomised controlled trial of early intervention in young children. As such, our results extend the findings from the smaller study of Estes and colleagues, with a shorter follow-up period of 2 years^11^ that suggested follow-up effects on autism symptoms. Notably, the PACT intervention is substantially less time intensive than is Estes and colleagues intervention (ESDM) in terms of therapist treatment hours. Taken together, these results are encouraging and provide evidence that sustained changes in autism symptoms can be possible after early intervention, something that has previously been regarded as difficult to achieve.

Our findings also have substantial implications for developmental science, given that a sustained effect of this type on targeted outcomes for a prolonged period after the end of treatment is very uncommon in developmental interventions. PACT is designed to work with parents to reduce autism symptoms through optimising naturalistic parent–child social communication in the home setting. The theoretical advantage of this approach over direct therapist–child therapy is that it has potential for change in the everyday life of the child, in which much social learning takes place, and might continue to have sustained effects after the end of the therapist-delivered intervention to the parent. Evidence from the initial trial mediation analysis^16^ supported this theoretical model of action by showing that it was the treatment effects on parent interaction style that were responsible for the positive child change during the treatment period. The current follow-up analysis shows a loss of this original treatment effect on parental synchrony over time, but maintenance of the effects on child communication and symptom-change. This finding suggests that the improvement in child communication and autism symptoms during treatment could have become self-sustaining during the years following the end of treatment, independent of the initial parental behavioural change that mediated them. This finding lends support to the theoretical rationale behind a developmental approach in which targeting of pivotal precursor social communication skills can lead to improvements in developmental trajectories, further supporting the notion that a parent-mediated approach can lead to sustained effects beyond the end of treatment. Further empirical study of the mechanism behind such maintained change is now needed.

Before the follow-up study, we hypothesised that the PACT intervention would lead to sustained reduction in restricted and repetitive behaviours of the child. Testing of this hypothesis in a prespecified analysis produced another important finding: the intervention, which was targeted at improving child social communication, also had marked cross-domain effects on the restricted and repetitive behaviours aspect of autism, seen both at endpoint^6^ and at follow-up, with the follow-up result reflected in ADOS CSS and parent-reported RBQ scores. We argue that this finding supports a developmental account of the emergence of restricted and repetitive behaviours as at least partly dependent on a lack of effective social communication during the development of individuals with autism. However, our related hypothesis that levels of child anxiety, which are often linked to levels of restricted and repetitive behaviours in autism,^17^ would also show a treatment effect at follow-up was refuted (figure 2).

Amelioration of the defining symptoms of autism is positive in its own right; but, in longitudinal autism cohort studies, ADOS symptom trajectories also predict concurrent adaptive functioning and later long-range adjustment through to young adulthood.^31^, ^32^ Estes and colleagues reported an effect on parent-rated adaptive function during their 2 year follow-up.^11^ Our measures designed to address this aspect of autism were blinded teacher-rating of adaptive function in school, and non-blinded reports by parents of child adaptive function, peer relationships, and prosocial behavior. Point estimates of treatment effect on these measures range from modest to substantial size in favour of treatment, but CIs for the effect sizes were wide and included null or negative effects in each case (figure 2). Consequently we cannot be sure at this time of the existence or extent of the effect of symptom changes on general adaptation. We found no evidence of an overall effect on comorbid mental health problems; the only apparent effect, a small point estimate of effect on anxiety or OCD symptoms, had a wide CI that included both some negative and positive effect. These findings suggest that, at the least, additional strategies will be needed if broader adaptive function and mental health in autism at later ages is to be further improved.

The high rate of follow-up of what was already the largest randomised intervention cohort of its kind to address core autism features represents a key strength of this study; follow-up ascertainment was achieved for almost 80% of the recruited sample at 82 months after initial randomisation. Key measures were assessed in a blinded manner and consistently across time-points, although parent reports of repetitive behaviours and peer functioning were at follow-up only. Between-group demographic imbalances in the follow-up sample were adjusted for in the analysis. We note that our inclusion criteria were for core-defined autism rather than broader autism spectrum disorder; we cannot be sure how our results would generalise to young children with less severe symptoms.

This study advances previous work by showing that a theoretically derived, developmentally targeted early intervention can have a sustained effect on autism symptom outcomes nearly 6 years after the end of treatment. In addition to replication, further research is needed to elucidate the developmental mechanisms behind such sustained change, as well as the extent of and barriers to wider developmental benefits, and the cost-effectiveness of such interventions over longer-term development.

On the basis of these results, we are now able to support the use of the PACT intervention for reducing symptoms of autism in young children, a revision of our initial view^6^ and consistent with the results of a subsequent UK NICE meta-analysis.^8^

## Figures and Tables

**Table 1:** Baseline and follow-up participants by treatment group

		**Baseline**		**Follow-up**	
		PACT intervention (n=77)	Treatment as usual (n=75)	PACT intervention (n=59)	Treatment as usual (n=62)
Sex					
	Male	71 (92%)	67 (89%)	57 (97%)	54 (87%)
	Female	6 (8%)	8 (11%)	2 (3%)	8 (13%)
Age (months)		44·7 (7·8)	45 (8·1)	127·3 (9·2)	127·2 (9·9)
Centre					
	London	26 (34%)	26 (35%)	20 (34%)	21 (34%)
	Manchester	26 (34%)	26 (35%)	21 (36%)	23 (37%)
	Newcastle	25 (32%)	23 (31%)	18 (31%)	18 (29%)
Both parents living at home?					
	Yes	60 (78%)	57 (76%)	48 (81%)	47 (76%)
	No	17 (22%)	18 (24%)	11 (19%)	15 (24%)
Parents' ethnic origin					
	Both white	46 (60%)	41 (55%)	34 (58%)	35 (56%)
	Mixed ethnicity parents	5 (6%)	9 (12%)	5 (8%)	8 (13%)
	Both non-white	26 (34%)	25 (33%)	20 (34%)	19 (31%)
Family size					
	Other children	1·0 (0·8)	1·1 (1·0)	1·1 (0·8)	1·1 (0·9)
	Adults	1·8 (0·4)	1·8 (0·5)	1·9 (0·4)	1·8 (0·5)
	Parental education*	65 (84%)	47 (63%)	52 (88%)	39 (63%)
Socioeconomic status					
	Manual	26 (34%)	31 (41%)	17 (29%)	27 (44%)
	Professional or administrative	51 (66%)	44 (59%)	42 (71%)	35 (56%)

Data are mean (SD) or n (%). PACT=preschool autism communication trial.

* Defined as one parent with qualifications after age 16 years.

**Table 2:** Outcome descriptive statistics

	**PACT intervention**	**Treatment as usual**
Autism symptoms (ADOS CSS*)		
Baseline	8·0 (1·4)	7·9 (1·4)
Post-treatment	6·7 (1·7)	7·3 (1·6)
Follow-up	7·3 (2·0)	7·8 (1·8)
**Child initiations (dyadic)**†		
Baseline	22·7% (18·8)	26·1% (18·7)
Mid-treatment	41·0% (21·8)	30·1% (18·8)
Post-treatment	34·0% (18·7)	27·2% (17·6)
Follow-up	30·1% (17·5)	26·7% (17·0)
**Language composite**‡		
Follow-up	84·8 (38·6)	80·0 (40·0)
**Parent synchrony (dyadic)**§		
Baseline	30·7% (14·2)	31·1% (16·0)
Mid-treatment	53·8% (20·2)	33·8% (14·5)
Post-treatment	53·0% (20·9)	33·4% (14·4)
Follow-up	44·4% (16·1)	43·1% (15·7)
**Conversation turns (dyadic)**¶		
Follow-up	28·3 (24·4)	26·2 (19·4)
**Teacher-rated adaptive behaviour**‖		
Follow-up	66·3 (21·3)	60·4 (16·6)
**Parent-rated adaptive behaviour**‖		
Baseline	65·3 (8·1)	65·5 (9·0)
Post-treatment	67·5 (13·0)	65·2 (12·2)
Follow-up	63·2 (14·8)	60·7 (11·3)
Social communication (SCQ score**)		
Follow-up total	27·4 (5·8)	29·0 (5·1)
**Repetitive behaviour (RBQ score**††**)**		
Sensory-motor (follow-up)	4·8 (3·4)	8·3 (4·2)
Sameness (follow-up)	7·1 (4·3)	11·6 (6·3)
**Strength and difficulties (SDQ score**‡‡**)**		
Peer problems (follow-up)	5·0 (2·4)	5·7 (1·7)
Prosocial (follow-up)	4·7 (2·9)	3·7 (2·7)
**Co-occuring disorder at follow-up (DAWBA**§§**)**		
Depression	2/50 (4%)	3/44 (7%)
Conduct/oppositional disorder	17/50 (34%)	17/44 (39%)
Hyperkinesis	6/50 (12%)	7/44 (16%)
Anxiety/OCD	12/50 (24%)	16/46 (35%)

Data are mean (SD) unless noted otherwise. PACT=preschool autism communication trial. ADOS CSS=Autism Diagnostic Observation Schedule Comparative Severity Score. DCMA=Dyadic Communication Measure for Autism. SCQ=Social Communication Questionnaire. RBQ=Repetitive Behaviour Questionnaire. SDQ=Strength and Difficulties Questionnaire. DAWBA=Development and Well-Being Assessment. ICD-10=International Statistical Classification of Diseases and Related Health Problems 10th Revision. OCD=obsessive compulsive disorder.

* Low ADOS CSS represents less severe autism symptoms.

† Untransformed proportion of child behaviours that were initiations in dyadic interaction with parent (DCMA).

‡ Language composite of six subscales.

§ Untransformed proportion of parent behaviours synchronous with child in dyadic interaction with child (DCMA).

¶ Untransformed number of conversational turns in dyadic interaction (DCMA).

‖ Teacher and parent Vineland Adaptive Behaviour Composite V scores.

** Parent-rated SCQ total score.

†† Parent-rated RBQ score, with scoring based on a factor analysis reported previously.^26^

‡‡ Parent-rated SDQ, where a low peer problems score represents less peer difficulties, and a high prosocial represents more prosocial behaviour.

§§ In the DAWBA, ICD-10 disorders were grouped into four categories: depression, conduct or oppositional disorders, hyperkinesis, and any anxiety or OCD; in cases of multiple disorders categories, the disorder with the highest likelihood level was used; data are presented as binary, with the cutpoint of greater than or equal to 50% for presence of disorder.
