# Supplementary material for: Parent-mediated social communication therapy for young children with autism (PACT): long-term follow-up of a randomised controlled trial
Source: Lancet. 2016 Nov 19;388(10059):2501–9. doi: 10.1016/S0140-6736(16)31229-6 (PMC5121131; doi:10.1016/S0140-6736(16)31229-6)
Supplement: Supplementary appendix [file mmc1.pdf]

THE LANCET

## Supplementary appendix

This appendix formed part of the original submission and has been peer reviewed. We post it as supplied by the authors.

Supplement to: Pickles A, Le Couteur A, Leadbitter K, et al. Parent-mediated social communication therapy for young children with autism (PACT): long-term follow-up of a randomised controlled trial. *Lancet* 2016; published online Oct 25. http://dx.doi.org/10.1016/S0140-6736(16)31229-6.

**Parent-mediated social communication therapy for young children with autism (PACT): long-term follow-up of a randomised controlled trial**

**Online Supplement**

Authors: Pickles A, Le Couteur A, Leadbitter K, Salomone E, Tobin H, Cole-Fletcher R, Gammer I, Lowry J, Vamvakas G, Byford S, Aldred C, Slonims V, McConachie H, Howlin P, Parr J, Charman T, Green J.

## Online Supplement

sMethods

sFigure 1 Language factor structural equation model

sTable 1 Treatment effect estimates following multiple imputation

sTable 2 ADOS-CSS Social Communication and Restricted Repetitive Behaviour sub-domain total effects

**sMethods**

*Analysis Plan*

This was specified prior to data-lock and unblinding. During analysis: (1) two secondary outcomes were dropped, Mutual Shared Attention from the DCMA interaction coding that had been suggested for descriptive reporting but on reflection was considered age-inappropriate and a family life score from a questionnaire for which published validation had not become available; (2) for the longitudinal models the Area-Under-the-Curve estimates were added as a means of summarizing treatment effect over the course of the study and multiple imputation and GEE/robust standard error estimation was used as an additional check as the robustness of the findings to model misspecification and missing data; (3) a proposed Venn-diagram spanning five categories of comorbid disorders at follow-up was dropped as being too disaggregated to be interpretable; (4) bootstrap standard errors were used for the confidence intervals of all effect estimates as their properties were likely to be superior to those based on standard asymptotics for many of the statistics presented.(5) The inclusion of dummy variables for centre effects.

*Language composite factor model*

The structure of the model is shown in eFigure 1. The model allowed the errors of the measures within each of the three assessment tools to be correlated, that is, measures 1 and 2, measures 3 and 4, and measures 5 and 6. This model fitted satisfactorily with a goodness of fit chi-square value of 48.513 with 31 degrees of freedom (p=0.023), an RMSEA value of 0.061, and a CFI value of 0.981. This model gave the p-value for the effect of treatment group on the latent language variable as p=0.436.

*Multiple Imputation*

Analyses reported in eTable 1 made use of the mi impute and mi estimate commands in Stata 13[1]. For each outcome variable any missing data for any of the repeated measures of that variable, the treatment group, the unbalanced variables and correlated baseline variables and ages at assessment were multiply imputed using chained equations. Reported estimates come from the application of Rubin's Rules[2] to combine estimates from analysis of the multiple datasets. Analysis models reported in eTable 1 paralleled those in the main paper except that with complete data the opportunity was taken to use model robust methods (GEE and robust standard errors) for the analysis of repeated measures.

### sFigure 1 Language factor SEM

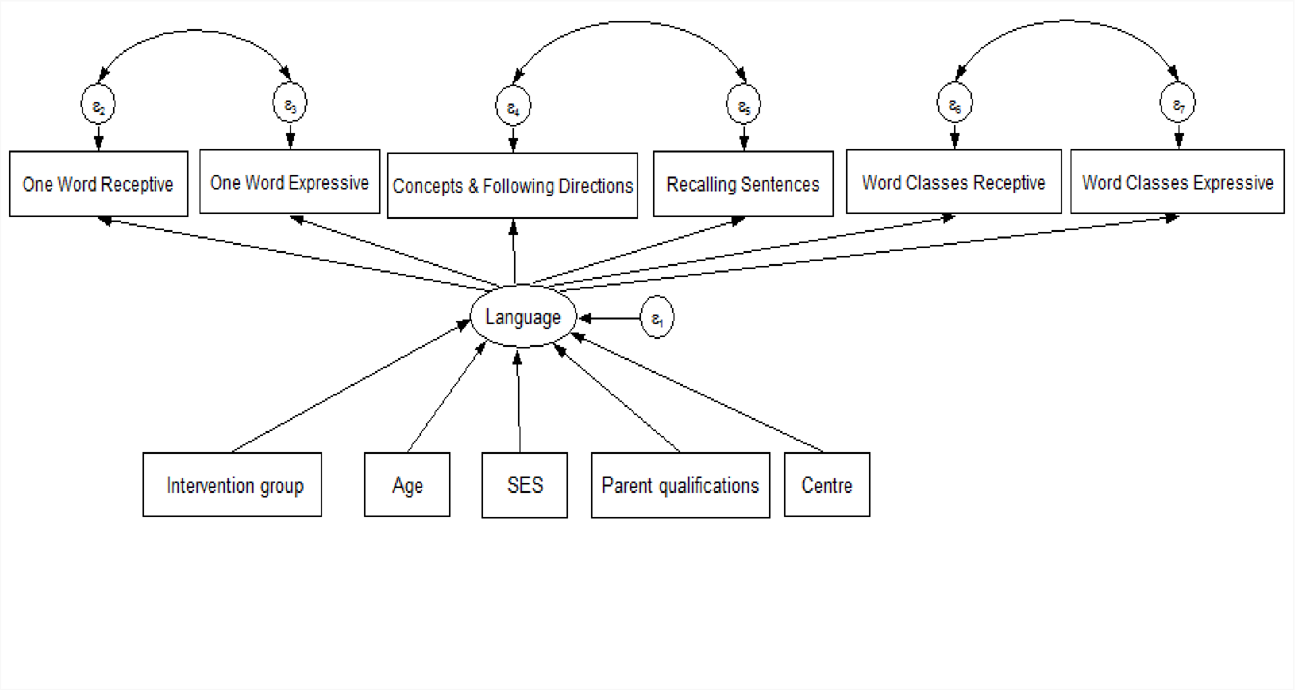


### sTable 1 Treatment effect estimates following multiple imputation

| **ADOS CSS**[i] | Log ordinal odds ratio (SE) | P-value | CIs | |
|---|---|---|---|---|
| Trt at Endpoint | -0.64 (0.30) | 0.03 | -1.22 | -0.06 |
| Trt at Follow-up | -0.82 (0.36) | 0.02 | -1.53 | -0.12 |
| **Child Initiations**[ii] | Logit proportions (SE) | P-value | CIs | |
| Trt at Midpoint | 0.72 (0.23) | 0.002 | 0.27 | 1.17 |
| Trt at Endpoint | 0.60 (0.23) | 0.009 | 0.15 | 1.05 |
| Trt at Follow-up | 0.51 (0.26) | 0.05 | 0.002 | 1.01 |
| **Parent Synchrony**[ii] | Logit proportions (SE) | P-value | CIs | |
| Trt at Midpoint | 0.87 (0.13) | >0.00 | 0.63 | 1.12 |
| Trt at Endpoint | 0.90 (0.14) | >0.00 | 0.62 | 1.18 |
| Trt at Follow-up | 0.14 (0.15) | 0.34 | -0.15 | 0.43 |
| **Conv. Turns**[ii] | Coefficient (SE) | P-value | CIs | |
| Trt at Follow-up | -0.05 (0.25) | 0.86 | -0.56 | -0.47 |

| | | | | |
|---|---|---|---|---|
| **Teacher Vineland ABC**[iii] | Coefficient (SE) | P-value | CIs | |
| Trt at Follow-up | 5.60 (3.44) | 0.11 | -1.22 | 12.41 |
| **Parent Vineland ABC**[iii] | Coefficient (SE) | P-value | CIs | |
| Trt at Endpoint | -2.57 (1.57) | 0.10 | -5.65 | 0.50 |
| Trt at Follow-up | -3.49 (1.89) | 0.07 | -7.20 | 0.23 |
| **SCQ**[iv] | Coefficient (SE) | P-value | CIs | |
| Trt at Follow-up | -2.50 (1.11) | 0.028 | -4.71 | -0.28 |
| **RBQ sensory-motor**[v] | Coefficient (SE) | P-value | CIs | |
| Trt at Follow-up | -3.55 (0.74) | 0.000 | -5.02 | -2.09 |
| **RBQ sameness**[v] | Coefficient (SE) | P-value | CIs | |
| Trt at Follow-up | -4.30 (1.10) | 0.000 | -6.49 | -2.11 |
| **SDQ peer problems**[vi] | Log ordered odds ratio (SE) | P-value | CIs | |
| Trt at Follow-up | -0.59 (0.36) | 0.11 | -1.31 | 0.13 |
| **SDQ prosocial**[vi] | Log ordered odds ratio (SE) | P-value | CIs | |
| Trt at Follow-up | 0.69 (0.36) | 0.06 | -0.02 | 1.39 |

[i] ADOS CSS Low score = less autism symptom severity: Estimate from ordinal logit with random intercept and robust standard errors

[ii] Dyadic Communication Measure for Autism (DCMA). Child communication initiation with parent, calculated as a proportion of all child acts, logit transformed for normality; parental synchronous response with child, calculated as the logit transformed proportion of all parent acts; conversational turns (follow-up only) between parent and child: Estimates from GEE with exchangeable correlation matrix and robust standard errors

[iii] Adaptive Behavior were the Teacher and Parent Vineland Adaptive Behavior Composite V scores: Parent estimate from GEE with exchangeable correlation matrix and robust standard errors; Teacher estimate from regression.

[iv] SCQ: Parent-rated Social Communication Questionnaire ; total score : Estimate from regression.

[v] RBQ: Parent-rated Repetitive Behaviour Questionnaire. Scoring was based on a factor analysis reported in Honey et al. (2012): Estimates from regressions.

[vi] SDQ: Parent-rated Strengths and Difficulties Questionnaire: ‘Peer Problems’ low score = less Peer Difficulties, ‘Prosocial’ high score = more prosocial behavior: estimates from ordinal logistic regression.

**sTable 2 ADOS Social Affective and Restricted and Repetitive sub-domains:[3] overall effects from repeated measures ordinal response model**

| Domain | Log-odds | 95% Bootstrap Confidence interval | P-value |
|---|---|---|---|
| Social-Affective | 0.324 | -0.071, 0.624 | .060 |
| Restricted & Repetitive | 0.820 | 0.119, 1.591 | .026 |

## References


1 StataCorp. Stata Statistical Software. In: Release 13. Texas: StataCorp LP; 2013

2 Rubin DB. Multiple Imputation for Non-response in Surveys. New York, Wiley & Sons. 1987

3 Hus V, Lord C. The autism diagnostic observation schedule, module 4: revised algorithm and standardised severity scores. J Autism Dev Disord. 2014 Aug;44(8):1996-2012.

# PACT 7-11

## Follow-Up to the Pre-school Autism Communication Trial

**PACT 7-11**

**STUDY PROTOCOL**

**Version 3 (22.11.13)**

CONTENTS

1. General Information

2. List of Protocol Amendments

3. Lay Person Summary

4. Scientific Justification and Background Information

5. Objectives

6. Study Design

7. Timeline

8. Procedures

9. Measures

10. Analysis

11. Data Handling and Record Keeping

12. Steering, Monitoring and Quality Assurance Procedures

13. Dissemination

14. Finance

15. Ethical Considerations and Approval

16. References

**1. GENERAL INFORMATION**

| | |
|---|---|
| Title | Follow-Up to the Pre-school Autism Communication Trial at 7-11 Years<br><br>**ISRCTN:**<br>**MRC REFERENCE:** MR/K005863/1 |
| **Co-ordinating Office** | ***The PACT 7-11 Office***<br>Room 4.321<br>Jean McFarlane Building<br>The University of Manchester<br>Oxford Road<br>Manchester<br>M13 9PL<br>Email: Kathy.leadbitter@manchester.ac.uk<br>Website: http://www.bbmh.manchester.ac.uk/pact/ |
| **Funders** | *Medical Research Council (research funding)*<br>MRC Reference: MR/K005863/1<br>20 Park Crescent<br>London,<br>W1B 1AL<br>Tel: 020 7670 5271 |
| **Sponsor and Monitor** | *University of Manchester*<br>Lynne Macrae<br>FMHS Research Office<br>3.53 Simon Building<br>University of Manchester<br>Oxford Road<br>Manchester<br>M13 9PL<br>Tel: 01612755436<br>Email: fmhsethicsapps@manchester.ac.uk |
| **Collaborating Institutions** | University of Manchester<br>Central Manchester University Hospitals NHS Foundation Trust<br>Stockport NHS Foundation Trust<br>University of Newcastle<br>Newcastle-upon-Tyne Hospitals NHS Foundation Trust<br>City Hospitals Sunderland NHS Foundation Trust<br>Northumberland Tyne & Wear NHS Foundation Trust<br>Guys & St Thomas' NHS Foundation Trust, London<br>Institute of Psychiatry, King's College London<br>Lewisham PCT<br>Southwark PCT<br>Lambeth PCT |

**Chief Investigator**

Prof Jonathan Green
Room 4.305
Jean McFarlane Building
The University of Manchester
Oxford Road
Manchester
M13 9PL
Tel: 0161 3067967
Email: jonathan.green@manchester.ac.uk

**Principal Investigators**

Dr Catherine Aldred
Specialist Consultant in Speech & Language Therapy
Stepping Hill Hospital
Poplar Grove
Stockport
SK2 7JE
Email: craldred@tiscali.co.uk

Professor Sarah Byford
Professor of Health Economics & Co-Deputy Director Centre for the Economics of Mental & Physical Health (CEMPH)
Institute of Psychiatry at King's College London
Box P024,
The David Goldberg Centre
De Crespigny Park
London
SE5 8AF
Tel: 020 7848 0043
Email: s.byford@kcl.ac.uk

Professor Tony Charman
Institute of Psychiatry at King's College London
Department of Psychology
Box PO77
Henry Wellcome Building
De Crespigny Park
Denmark Hill
London
SE5 8AF
Tel: 0207 848 5038
Email: tony.charman@kcl.ac.uk

Professor Pat Howlin
Professor of Clinical Psychology
Dept of Psychology
Institute of Psychiatry
Denmark Hill
London SE5 8AF
Tel: 0207 848 0815
Email: patricia.howlin@kcl.ac.uk

Professor Ann Le Couteur
Professor of Child & Adolescent Psychiatry
University of Newcastle
Institute of Health & Society
Sir James Spence Institute
Royal Victoria Infirmary

Queen Victoria Rd
Newcastle upon Tyne
NE1 4LP
Tel: 0191 282 1384
Email: A.S.le-Couteur@newcastle.ac.uk

Professor Helen McConachie
Professor of Child Clinical Psychology
Institute of Health and Society
Newcastle University
Sir James Spence Institute 3rd floor
Royal Victoria Infirmary
Newcastle upon Tyne NE1 4LP
Tel: 0191 282 1396
Email: helen.mcconachie@newcastle.ac.uk

Dr Jeremy Parr
Clinical Senior Lecturer & Honorary Consultant in Paediatric Neurodisability
Sir James Spence Institute
Royal Victoria Infirmary
Queen Victoria Rd
Newcastle Upon Tyne
NE1 4LP
Tel: 0191 282 4725
Email: jeremy.parr@ncl.ac.uk

Professor Andrew Pickles
Institute of Psychiatry
Dept of Biostatistics
King's College London
De Crespigny Park
London
SE5 8AF
Tel: 0207 848 0724
Email: andrew.pickles@kcl.ac.uk

Dr Vicky Slonims
Clinical Lead Speech and Language Therapist
Honorary Senior Lecturer (Kings College London)
Children's Neurosciences Centre,
Newcomen Centre at St Thomas',
Staircase D South Wing,
St Thomas' Hospital,
Westminster Bridge Road
London SE1 7EH
Tel: 020 7188 4648
Email: Vicky.Slonims@gstt.nhs.uk

**Study Statistician**

Professor Andrew Pickles
Institute of Psychiatry
King's College London
De Crespigny Park
London
SE5 8AF

**Research Team**

Dr Kathy Leadbitter (lead researcher)
Rachel Cole-Fletcher

| | |
|---|---|
| | Isobel Gammer<br>Erica Salomone<br>Hannah Tobin<br>Jessica Lowry |
| **Steering Committee** | Professor Jonathan Green, Chief Investigator, University of Manchester<br>Professor Eric Taylor, Consultant Psychiatrist, Institute of Psychiatry; Former Chair of PACT Steering Committee<br>Mr Richard Mills, National Autistic Society Director of Research<br>Professor Andrew Pickles, Statistician, Institute of Psychiatry<br>Lindsay Stairs (Parent Representative)<br>Bridget Gilchrist (Parent Representative) |

**Mental Health Research Network (MHRN)**
**Trial adopted on the network** June 2013

**PACT 7-11 will be conducted in accordance with the principles of GCP and applicable UK regulatory requirements**

## 2. PROTCOL AMENDMENTS

The following changes have been made to the protocol.

| *Version Number* | *Date* | *Section* | *Amendment* |
|---|---|---|---|
| v.2 | 05.09.13 | General Information<br>Timeline<br>Assessment Procedures and Measures | Updated personnel details<br>Updated timescales<br>Updated information about parent wellbeing measure<br>New information about MIPO Playground Observation Measure |
| v. 3 | 22.11.13 | Assessment Procedures and Measures | New information about Social Communication Questionnaire and Repetitive Behaviour Questionnaire, replacing information about Autism Diagnostic Interview- Revised |

## 3. LAY PERSON SUMMARY

Autism is a severe and lifelong developmental disorder affecting about 1% of children. Families of a child with autism often feel a huge strain due to the child's communication and behavioural difficulties and increased care needs. The Pre-school Autism Communication Trial (PACT) was the largest trial to date of an early intervention to help parents communicate better with their child with autism. This study (Green et al., 2010) showed that parents who took part in the intervention were successful at adapting their style of interacting with their child, and in turn the child communicated more with the parent. However the intervention did not help children to interact better with adults outside the family, that is, the communication improvements did not seem to generalize.

The initial PACT follow-up of 13 months was relatively short. Little is known generally about the longer term effects of early intervention. Therefore in this current study we wish to follow-up the PACT children about 4 years on (aged 7 to 11) to see how our intervention has affected longer

term outcomes. We will be looking at the child's language and communication, other symptoms of autism, and difficulties of social integration and mental health problems. We are interested in parent perceptions of their family life and whether parents who took part in the intervention feel more confident and optimistic than those who did not.

This study will help us understand, for the first time, the impact of early intervention on later child communication, the presentation of autism, and family life, school adjustment and family need for services. This will give an indication of any necessary additional intervention required to maximise the best developmental outcomes in autism. The findings will potentially impact the way autism intervention is organised and methods of supporting families as their children grow older.

## 4. SCIENTIFIC JUSTIFICATION AND BACKGROUND INFORMATION

Autism is a severe and persistent developmental disorder, starting in childhood and often extending throughout life and affecting about 1% of children in the community. Autism is diagnosed from about 2 years of age onwards. Parents find adjustment to diagnosis and the management of their child with autism very challenging; use of health, social care, and specialist education resources increases progressively through the pre-school and early school years with costs of supporting children with autistic spectrum disorders in the UK estimated at £2.7 billion per year. However, the additional impact of autism on families is probably far higher than this in terms of intense parental involvement and stretching of resources to care for other children and to sustain employment. Rates of family breakdown are higher than the average.

Early intervention is thought to be most beneficial and there is increasing evidence that programmes of appropriate advice and training improve child communication and behaviour. However, early intervention trials for children with autism are typically time-limited, with virtually no substantive follow-up studies to test longer-term outcomes (Charman, 2011) despite autism being a lifelong condition needing treatment organised within a longer-term developmental context (Koegel et al., 2010).

The MRC Preschool Autism Communication Trial (PACT) was a 3-site 2-arm parallel group randomised trial (n=152) of a targeted, developmental theory-based, communication-focused intervention for pre-school children with autism (age 2 years to 4 years 11 months). It is the largest psychosocial trial for children with autism to date, recognised as setting a new benchmark in autism intervention research (Spence et al., 2010). Internal validity was outstanding with very low attrition to endpoint. The sample was geographically and demographically representative within England. After 7 months of intervention there was evidence of a substantive treatment effect in enhancing these targeted developmental processes, and these effects were sustained at 13 month endpoint, with modest attenuated effects on generalized outcomes.

This study (PACT 7-11) will be an innovative and important follow-up of this valuable, internationally-recognised and well-characterised cohort of preschool children with autism into middle childhood - a relatively neglected stage of developmental research in autism. Our initial follow up was only at the end of the 13 month intervention – a very short time in the context of development for a child with autism. In this current study we wish to follow-up the same children about 4-6 years further on to see how our early intervention has affected longer term developmental outcomes. We will be doing the follow-up in the mid-school years (aged 7-11 years) which is a key time in the development of a child with autism, when many associated difficulties of social integration, and mental health problems such as anxiety, come to the fore.

The study will enable us to consider, in depth, what factors earlier in life lead to difficulties and secondary health and mental health problems in middle childhood. It will also allow us to identify whether early preschool intervention can prevent later problems and provide information for future design of middle childhood interventions. In addition, it will provide valuable information on the longer-term effects of early intervention on child and family functioning, including the burden on carers, and service use. Developmental studies in autism show that many of these characteristics measured in mid-childhood are predictive of outcomes through adolescence into adulthood. Thus,

knowing the medium-term effects (and limits) of a treatment such as the PACT intervention holds some promise to reduce burden to the individual, their family and society across the lifespan in the future.

PACT was designed from the outset as a 'developmental experiment' (Green et al., 2006), using the RCT to test both outcomes and causal developmental models in early autism. It focused on detailed evaluation of putative mediation and causal processes, using theory-based measures of developmental outcomes within a pre-specified analysis. Our proposed study follows the logic of this approach with a developmental follow-up that in effect completes a 'hybrid trial' design (Howe et al., 2002), combining a parallel group random allocation, an intervention arm targeting developmentally-key processes, and an observational follow-up of two resulting parallel developmental cohorts. This is a unique way of testing causal influence in development (Green et al., 2008; Howe et al., 2002; Long et al., 2008). This proposal thus builds on the strengths of the PACT cohort and our team's expertise in trials, autism developmental science, longitudinal cohort studies and methodology.

## 5. STUDY OBJECTIVES

### *5.1 Primary Objectives*

The PACT 7-11 study is designed to address three central questions:

1) Are the changes in developmental processes achieved in PACT sustained at 4-6 year follow up?

This question is a priority because it addresses an important issue of whether time-limited interventions of this kind are sufficient in a persistent developmental disorder, or whether longer or repeated phased interventions are necessary. The question will be answered through follow-up ascertainment of at least 80% of the original PACT Trial sample after 4-6 years in middle childhood, repeating comparable measures from the original trial, including:

a. parental synchronous communication with the child (the targeted developmental process in the intervention)
b. child communication initiations with parent
c. measures of child communication and child and family adaptive functioning.

2) Does the enhancement of these early developmental variables result in later positive developmental outcomes in areas expected from theory?

Theory and independent longitudinal observation studies suggest that changing the early variables may impact on a number of specific longer-term outcomes including specifically child language and communication. The follow-up will also test generalised symptom and adaptation outcomes, which show modest effects at trial endpoint, as to whether the endpoint occurred before generalisation occurred. These hypotheses will be tested at follow-up by measuring:
a. Child communication and language outcomes
b. Autism symptoms
c. Parent reported outcomes in child development, parental and family functioning
d. Burden of parental care and service use

3) Can we use a treatment trial of this kind as a 'developmental experiment' to illuminate basic questions in the development science of autism?

The unique strength of this proposal is that the initial randomisation in the PACT trial allows for a causal interpretation of subsequent effects, something that does not apply in observational longitudinal studies. We will thus use the developmental follow-up of randomly allocated parallel cohorts and application of methodological innovations developed within the trial team to test a number of basic science hypotheses in autism:

a. The enhanced parental synchronous communication with their child produced in the PACT intervention arm will (as predicted by theory and previous evidence) differentially enhance later child language and communication abilities compared to the non-PACT treated arm.

b. The enhanced parent / child dyadic communication achieved in the intervention arm will have differential secondary impact on downstream autism symptoms, both in social communication and repetitive and stereotyped behaviour domains.

c. The impact of PACT intervention in self-report family functioning seen at trial endpoint, will be sustained at follow-up and have downstream effect on child development, predicted by theory, in terms of improved social adaptation and decreased co-morbid psychopathology.

d. Test of potential long-term moderators of intervention outcomes in development. These include baseline variables identified in the initial PACT trial, including SES, autism severity, initial IQ, and traits of the 'broader autism phenotype' in parents undertaking the intervention.

### *5.2 Secondary objectives*

There are four main secondary research objectives.

1) Reduced anxiety. Repetitive, ritualistic and stereotyped behaviours, especially the insistence on sameness and intense special interests, tend to increase with age and can have serious impact in middle childhood. Coupled with the impairments in social communication, these can lead to elevated levels of anxiety in children with autism. If the PACT therapy leads to improved communication and reduced repetitive, ritualistic and stereotyped behaviours, does this in turn lead to reduced levels of anxiety?

2) Fractionability. There is continuing scientific debate on the 'fractionability' of the components of the autism spectrum, that is, whether the social communication and the repetitive, ritualistic and stereotyped behaviour domains occur along separable developmental lines that happen to co-exist in autism. In testing the questions above, we will contribute to this debate by asking whether the social communication intervention showed a significant longitudinal effect on repetitive, ritualistic and stereotyped behaviours.

3) Family wellbeing and child adaptation. A key rationale for parent-mediated intervention is its potential to bring about benefits for the family as a whole. The PACT trial found that parents who had taken part in the therapy reported improved levels of adaptation in their child, compared to parents not involved in the therapy. They also reported relatively higher levels of family wellbeing. The follow-up study will test whether these effects have been sustained over time. It will also assess whether these improvements have longer-term implications for the child's mental health, the intensity of care the parent needs to provide for the child and also, the amount of support services families require.

4) Broader autism phenotype. Family association and genetic studies have shown that some family members of individuals with autism show low-levels of autism traits. The PACT intervention works through facilitating the parent to interact with their child in a more sensitive, responsive and communication-focussed way. It may be that parental traits of rigidity, lack of empathy and resistance to change might affect their ability to take part in and deliver the PACT therapy effectively. This would be important for us to understand. The initial study used a brief measure of these traits and found that they were associated with the extent to which parents taking part in the therapy were able to become more responsive to their child. In this study we aim to carry out a more in-depth assessment to assess whether, over time, the degree of these traits influences whether the PACT therapy has a long-term effect on the child's development.

## 6. STUDY DESIGN

This is a follow-up study of the Pre-school Autism Communication Trial. As such it takes the form of a "hybrid trial" design, i.e. combining a random allocation intervention trial with a longitudinal observational follow-up. This will allow us to test hypotheses, informed by the findings from the original trial combined with evidence from previous longitudinal cohort studies, about expected developmental outcomes from pre-school to the early school years.

### *6.1 Methods to minimise bias*

The research assistants and associates and their supervising senior research staff will be kept blind to the original treatment allocation of the PACT families. This will ensure that they are free from bias in data collection. Parents (and children where applicable) will be asked not to disclose their allocation to the research staff. The need for blinding of researchers and the request for non-disclosure was emphasised in the PACT trial and so parents already have some experience of this process. In the event of unblinding, the researcher will ask a colleague who has not been unblinded to code the ADOS and DCMA assessments (which are video-recorded and potentially subject to bias). Treatment data from the PACT trial will be kept separately to research data and will not be accessible to the research team.

### *6.2 Inclusion criteria*

The following inclusion criteria applied to the original PACT trial:

- From Age 2 to 4yr 11 months at baseline ADOS assessment.
- Fulfilling diagnostic criteria for core autistic disorder (AD) on both social and communication domains of the ADOS and on two out of the three functional domains of the ADI-R.
- Child has 13 months or more equivalent level in general (non-language) development.
- The family have sufficient spoken English at home which is adequate to allow them to participate in this communication based intervention.
- Neither child nor parent has long term severe hearing or visual impairment
- No current clinically severe psychiatric illness in parents.

The inclusion criterion for this follow-up study is: any child and parent(s) who participated in the original PACT trial.

### *6.3 Exclusion criteria*

The following exclusion criteria applied to the original PACT trial:

- Epilepsy requiring regular medication
- Twins

A further exclusion criterion will apply in the follow-up study:

- death of child (who was the original participant)

The effect of other major family adverse events on participation in the follow-up study will be considered sensitively and on a case-by-case basis. Participation will be discussed with the family and/or relevant professionals where appropriate. Such events would include: death of parent; significant mental/physical illness in child or parent; emigration outside of UK; child no longer living with original caregivers or in local authority care. If in any doubt of the appropriateness of inclusion/ exclusion, researchers will discuss the issues with their supervisor (who will be a senior clinical academic).

## 7. TIMELINE

| | |
|---|---|
| Nov 2012 - June 2013: | Start up tasks |
| Feb 2013 - June 2013: | Staff training |
| Feb 2013 - Jan 2014: | Tracing and consent |
| July 2013 - July 2014: | Fieldwork assessments |
| Feb 2014 - July 2014: | Coding and data entry |

May 2014 - Oct 2014: Data cleaning and analysis
Aug 2014 - Jan 2015: Write up and dissemination

# 8. PROCEDURES

## *8.1 Procedures for tracing and contacting participants*

Detailed thought was given to the procedure for contacting families to ensure that all families are reached and invited to participate, whilst at the same time avoiding intrusive contact and undue pressure to take part. The team has had ongoing contact with all PACT families since the end of the trial, through newsletter and postcard contact. We therefore have up-to-date contact details for many of the families. Families gave consent during initial trial for re-contacting in the event of a follow-up study and also gave the details of a family member and permission to contact this individual if the family were no longer contactable. Families have been informed since the original RCT (via the trial-closure newsletter) that there was a possibility that we would get back in touch for a follow-up study.

Initial contact for this study will come from the research team. We will start with general means of contact (newsletter, mailshot) so that families are expecting our contact and follow this with more direct contact (phone-call, email). Indirect means of contacting the family (via a relative or GP) will take place only once all other routes have been exhausted. Once contacted, if parents communicate an unwillingness to participate in the study for whatever reason, the researcher will have a very brief discussion to ascertain that there are no barriers to participation that could be easily overcome. They will then ask whether the family would still like to be (a) kept informed of the progress of the research, (b) contacted in the event of future studies. These decisions will be documented. The researcher will then thank the family for their involvement to date and that will terminate the contact for this study.

The following tracing and contacting procedure will be followed.

1. For each family, we will obtain the most up-to-date contact details we have on record from participant files, change of address cards, and databases.
2. We will maintain a central and stable address, phone number and email address for each site team. We will set up an answer phone that is always on with a clear message, that is checked regularly.
3. Prior to the recruitment phase we will send a newsletter to all families. On envelopes, we will state "If undelivered or not known at this address please return to…" with our return address. We will include a return slip with a pre-paid envelope for the family to return to opt-out or notify us of any change in details. We will invite families to check the website and to get in touch by phone or email, to opt in or out, to ask any questions, or to inform us of changes of details or circumstances.
4. We will send a letter to each child's GP to receive up-to-date address and phone number details and to provide us with information about any adverse family events.
5. At the start of the recruitment phase we will carry out a mailshot. This will be the formal invitation to take part. We will provide a slip with prepaid envelope to return with change of phone number/ other details. We will invite families to call or email us. We will inform families that we will be in touch soon by phone.
6. We will then call families' phone number (landline and mobile), trying at different times of the day and weekends if necessary. We will leave a message on the answer phone to call back.
7. We will try and contact by email, if we have the address.
8. If the above efforts at contact fail, we will call the family relative's phone numbers (recorded as part of PACT demographics interview and given consent to contact in the event of a follow-up study). We will explain to the family member about the study and ask them to pass on the families' contact details. If they are not happy to do this, we will ask them to pass on our details to the family.
9. In the event of failure to contact using the above methods, we will use other tracing activities as follows.
   a) Contact the child's GP directly, requesting contact details

b) Go through clinical teams (paediatrician, CAMHS, SALT etc)
c) Go through the child's school (if known)/ local education authority
d) Speak to previous PACT team members as they may recall other leads to follow or may have heard updates on the family since the trial
e) NHS Central Operations Management (they are able to match up names and NHS numbers to the new GP practice)/ E-health. We already have records of the NHS numbers of all children in the study so these can be used for this purpose.

***8.2 Consent and assent***

From initial contact participants will be allowed as much time as they need to decide whether or not to take part. At initial telephone contact we will discuss the study and make sure the parent has received and read the information sheet. If the parent is happy to go ahead there would be a bare minimum of 24 hours before the researcher visits to obtain written consent. If the parent needs time to make their decision or discuss with others, they will be given as much time as necessary, within the timescales of the data collection phase.

Written informed consent will be sought from parents after they have read the participant information sheet, discussed the study with the researcher and had ample opportunity to discuss the study with others and to make their decision. The researcher will encourage and facilitate discussion about the purpose and nature of the research and its benefits and burdens. The researcher will explicitly state in initial contact with the family that participation is voluntary. At no point will research staff put pressure on individuals to participate. Separate written consent will be obtained to make videorecordings. This consent form will specify that video material will be stored securely and will not be shown to individuals outside of the PACT team without further written consent.

There is no particular reason why any of the PACT parents should not be able to make informed consent. If, however, the individual seeking consent has reason to question this, they will discuss this with their supervisor.

The PACT participating children are unable to make informed consent due to their young age and, in some cases, because of their communication and learning disability. The children in this study will vary greatly in terms of their understanding and their ability to make and communicate decisions. We will therefore produce two information sheets to inform them about their involvement in the research. These sheets are based on information sheets our team has produced for other studies involving children with autism in this age range.

The first will be for children with lower levels of understanding of spoken and written language. This leaflet will contain mainly pictures, for example, a photograph of the researcher, where the research will be carried out, the activities that the child will engage in (e.g. toys they will be asked to play with). Alongside the photographs will be very simple descriptions that the parent and/or researcher can read to the child. A second information sheet will be produced for children with higher levels of understanding. This will also contain photographs but will contain a more detailed description of the research purposes and methods, which can be read to the child or the child can read for him/herself. Researchers will make the decision with advice from the parent about which information sheet is most appropriate for the child.

Assent will be assessed in two different ways. The first way is through an assent form. There will be two assent forms, to be used alongside the two information sheets (described above). In consultation with parent and upon meeting the child we will make a decision about whether to ask the child to complete an assent form at the start of the child assessments. The assent forms will contain tick boxes to show their agreement and a space to write their name. The second way is through the child's communication. Children with lower levels of understanding may not be able to understand or communicate assent in a formal way. However, researchers will be sensitive to signs that the child does not give assent, through their actions, expressions and behaviour.

***8.3 Assessment Procedures***

1. Initial contact

Once we have made contact with the parent(s) we will introduce ourselves and explain the nature of the follow-up study and what will be involved. We will invite the parent(s) to ask any questions they might have. We will send the Participant Information Sheet to the family, if they have not already received it. If the parent(s) is interested in taking part, we will arrange a home visit.

2. Home visit

The researcher will visit the parent(s) at home (or in the clinic if they prefer). The researcher will discuss the study in more depth, make sure the parent has read and understood the Participant Information Sheet, and answer any questions the parent has. The parent will be asked to read and sign the consent form and the video-consent form. The researcher will then carry out the following interviews with the parent:
- Family information and demographics questionnaire.
- Social Communication Questionnaire (Lifetime and Current Forms)
- Repetitive Behaviour Questionnaire (Long Form)
- The Vineland Adaptive Behaviour Scales, 2nd Edition (VABS-II).
- The Child and Adolescent Service Use Schedule (CA-SUS).
- The Family History Interview - Subject version (FHI-S)

If it is not possible to complete all the assessments within this visit, further home or clinic visits will be arranged with the family in order to ensure completion.

At the end of the home visit, the researcher will introduce the questionnaires and time diary to the parent and ask them to complete and return them to the researcher at the clinic visit.

The researcher will explain to the parent what will happen in the clinic visit. They will discuss which information sheet to leave for the child and provide a copy, asking the parent to go through it with the child prior to the visit. The researcher will also discuss with the parent how the child will be able to communicate assent.

3. Questionnaires
Between the home and clinic visit the parent will be asked to complete the following paper and online questionnaires:

- The Family Life Questionnaire
- Development and Well Being Assessment (DAWBA)
- Parent Wellbeing Questionnaire
- Parental Care Time Diary

If there are any difficulties with the parent filling these in independently, the researcher will sit with the parent and assist with issues of literacy or understanding the questions, whilst being certain not to influence the parent's responses. If the parent does not have access to the internet, the DAWBA will be conducted as an interview, in person or over the telephone, and the responses uploaded by the researcher.

4. Clinic visit (parent(s) and child)

The parent(s) and child will then attend the clinic setting. This will be a university assessment room or a clinic room in a hospital or health centre in their locality.

The researcher will go through the information sheet and assent form with the child and take written assent if applicable. Then the researcher will carry out the following assessments, which will be video-recorded for later coding and reliability purposes:

- Autism Diagnostic Observation Schedule (module 1, 2, or 3 depending on ability level of child)

- Clinical Evaluation of Language Fundamentals - Fourth Edition UK (CELF-4 UK: core subtests of expressive and receptive Language)

- Receptive and Expressive One Word Picture Vocabulary Test (REOWPVT)

- BAS-III: the Non-verbal composite (4 subtests: Recall of designs, Pattern construction, Matrices, Quantitative reasoning) or the Early Years Non-verbal subscales

- Parent-Child interaction, coded at a later point by the researcher using The Dyadic Communication Measure for Autism (DCMA).

If it is not possible to complete all the assessments within this visit, further home or clinic visits will be arranged with the family in order to ensure completion. All appointments will be offered at a time convenient to the family, including early evenings, weekends and school holidays if necessary.

5. School visit

With consent from the parent, the researcher will contact the child's school and explain the study and invite the child's teacher to help us. If they agree, the researcher will visit the school and administer the following assessments.

- Vineland-II Teacher Rating Form
- MIPO Playground Observation

6. Post-assessment

Upon completion of the assessments, the family will be sent a report summarising the parent interviews and child assessments.

All travel expenses incurred by participants as a direct consequence of taking part in the study will be reimbursed. The main expense will be expenses to attend clinic appointments. This will constitute mileage and parking allowance, refund of public transport tickets, or the reimbursement of taxi journeys on occasion (if it is difficult for the family to attend by any other means). These expenses will be paid in accordance with local site procedures for payment of expenses to research volunteers.

All participating families will receive a £30 high street gift voucher in a thank you card at the end of their participation in the study. This is a token of appreciation for their time and efforts with the assessments and their continued involvement in the research programme. This amount reflects the amount of time families will need to dedicate to the study and is in line with other studies run by our team, which have found the amount to be appropriate.

## 9. MEASURES

### *9.1 Primary outcome measures*

The primary outcomes measures are:

Autism Symptom Outcomes
- Autism Diagnostic Observation Schedule (ADOS) symptom severity

Language Outcomes - standardised scores of receptive and expressive language
- Clinical Evaluation of Language Fundamentals - Fourth Edition UK
- Receptive/ Expressive One Word Picture Vocabulary Tests - Fourth Edition

Family functioning and parental wellbeing

-Family Life Questionnaire
- Parent Wellbeing Questionnaire

***9.2 Secondary Outcomes measures***
The secondary outcome measures are:

Adaptive Behaviour Outcomes
-Vineland Adaptive Behaviour Scales, Second Edition
-Vineland Adaptive Behaviour Scales, Teacher Rating Form, Second Edition
- Manchester Inventory for Playground Observation (MIPO)

Family Service Use and Burden of Care
-Child and Adolescent Service Use Schedule (CASUS)
-Parental Care Time Diary

Child Mental Health
-Development and Wellbeing Assessment (DAWBA)

9.3 Moderators/ Mediators

Developmental Quotient
-British Ability Scales-III

Broader Autism Phenotype
-Family History Interview

***9.3 Description of Measures***

***9.3.1 Family and demographic information***

The family information and demographics questionnaire is a bespoke questionnaire designed to obtain up-to-date information about contact details, family/ household composition, and parental employment and education. It is researcher-administered and takes about 10 minutes to complete. This information is used to make summary statements about the demographics of the sample and compare the two groups (from the original randomisation process) on key statistics.

***9.3.2 Autism symptom outcomes***

The following three assessments are outcome measures of autism symptom severity.

**Social Communication Questionnaire** (SCQ; Rutter, Bailey, Berument, Lord & Pickles, 2003)

This brief instrument is used to evaluate the communication skills and social functioning in children who may have autism spectrum disorders. It is available in two forms—Lifetime and Current—each composed of just 40 yes-or-no questions. Each form is completed by a parent or other primary caregiver in less than 10 minutes.

The questions cover the following content areas:
- Language use
- Non-verbal communication
- Social development
- Play
- Interests and behaviours

The *Lifetime Form* focuses on the child's entire developmental history, providing a Total Score that is interpreted in relation to specific cut-off points. The *Current Form* looks at the child's behaviour over the most recent 3-month period. It produces results that can be helpful in the measurement of change over time.

The SCQ content parallels that of the ADI-R, and the agreement between SCQ and ADI-R scores is high and substantially unaffected by age, gender, language level, and performance IQ. This indicates that the SCQ is a valid screener, providing a reasonable picture of symptom severity.

**The Repetitive Behaviour Questionnaire** (RBQ Long Form; Honey et al., 2012)

The RBQ is a 33-item questionnaire short-form of the repetitive behaviour interview [RBI] (Turner, 1995). Twenty-nine items examine specific repetitive behaviours and parents are required to rate these for severity or frequency on a 3 or 4 point likert scale dependent upon the behaviour. Behaviours examined include repetitive movements, sameness behaviour, repetitive use of language and circumscribed interests. Four additional items include a summary item which examines a child's overall variety of interests, and three qualitative items about the age repetitive behaviours emerged and the most problematic and noticeable behaviours. The RBQ has been used in 3 published studies of repetitive behaviour in children with ASD, OCD and language delay (Barrett et al., 2004, Zandt et al., 2007 and Zandt et al., 2009) aged between 4 and 16 years. The form can be completed in approximately 10-15 minutes.

**Autism Diagnostic Observation Schedule-2** (ADOS2; Lord, Rutter, DiLavore, Risi, Gotham & Bishop, 2012)

This is a gold-standard direct observation measure of autism symptoms. It consists of a structured play-based assessment between researcher and child, videotaped for independent coding within domains of reciprocal social interaction, communication and repetitive and stereotyped behaviours. The ADOS includes four modules, each requiring 30-45 minutes to administer. One module is administered to each child, depending on his or her expressive language level and chronological age. The ADOS was used at Baseline and Endpoint assessment in the PACT study. In the follow-up we will be using newly-published severity and change additions.

***9.3.3 Language and communication outcomes***

The **Clinical Evaluation of Language Fundamentals - Fourth Edition UK** (CELF-4 UK; Semel et al., 2006) is a standardised assessment of receptive and expressive language abilities and difficulties. It assesses four aspects of language: morphology and syntax, semantics, pragmatics, and phonological awareness. This assessment will be administered to children with an estimated language level of five years or above. We will administer the core subtests of expressive and receptive Language and this will take 20-30 minutes to administer.

**Receptive and Expressive One Word Picture Vocabulary Test, Fourth Edition** (REOWPVT-4; Brownell et al., 2010). This is a brief pictorial standardised assessment of language comprehension and vocabulary. Children are asked to match a spoken word to an image or to name an object, action or concept from an image. It provides age-equivalents from two years into adulthood and takes 15-20 minutes to complete.

***9.3.4 Non-verbal IQ***

**The British Ability Scales –Third Edition (BAS-III)** is a standardised battery for assessing children's cognitive functioning. We will use the "non-verbal composite" which provides a standardised measure of non-verbal IQ. This consists of four subtests: Recall of designs, Pattern construction, Matrices, and Quantitative reasoning. For lower-functioning participants we will administer the Early Years Non-verbal subscales. These provide an age equivalence down to three years. The assessment will be administered by the researcher and takes around 30-45 minutes.

***9.3.5 Family functioning, adaptation and mental health outcomes***

The **Vineland Adaptive Behaviour Scales, 2nd Edition** (VABS-II; Sparrow et al., 2005) asks questions about the child's adaptive behaviour, that is, the personal and social skills needed for everyday living. The questionnaire is administered to the parent by the researcher using a semi-

structured interview format. There are five domains: communication, daily living skills, socialisation, motor skills and maladaptive behaviour. This takes about 20-60 minutes to complete.

The **Vineland Adaptive Behaviour Scales, 2nd Edition** (VABS-II; Sparrow et al., 2005) Teacher Rating Form assesses adaptive behaviour for pupils in school. This form uses a questionnaire format completed by the teacher or teaching assistant. The Teacher Rating Form contains the same Domains as the Survey Forms but covers content that a teacher would observe in a classroom setting. It takes around 20 minutes to complete.

The **Family Life Questionnaire** (Green et al., in prep) is a parental report across domains of parental confidence and self-efficacy, family functioning, child development and behaviour. This was developed for and used in the PACT Trial. It consists of statements about family life and parents tick boxes to indicate how often the statement applies to them. It takes 5-10 minutes to complete.

**The Development and Well Being Questionnaire** (DAWBA; Goodman et al., 2000) is a parental online rating of psychopathology and is used to identify important comorbidity (anxiety, ADHD, conduct and oppositional defiant disorder) in middle childhood. The parent will be asked to complete this online to provide information about comorbidity in the child. It takes about an hour to complete. If there are difficulties in completing this online, it will be administered as an interview over the phone, in the clinic or at home.

The **Parent Wellbeing Questionnaire** is a short tick-box questionnaire consisting of 26 items. It asked the parents about positive and negative aspects of their wellbeing. It comprises the General Health Questionnaire-12 and the Warwick and Edinburgh Mental Wellbeing Survey. Parents will be asked to complete this on a paper form. It takes about 5-10 minutes. This will provide a measure of parental health and wellbeing.

The **Manchester Inventory for Playground Observation** (MIPO) is a 10-minute observation of children's naturalistic play, interaction and behaviour within the school playground. A member of the research team not known to the child attends the school and observes the child and other children discretely during outdoor play-time. Behaviours of interest are noted with pen and paper and coded following the observation.

***9.3.6 Parent child interaction***

Parent and child are asked to play together for 15 minutes in a naturalistic way with a standardised set of materials. This interaction is video-recorded and coded at a later date using the **Dyadic Communication Measure for Autism** (DCMA; Aldred et al., 2011), an objective measure of parent-child dyadic communicative behaviours, which showed sensitivity to change and measured mediation in PACT.

***9.3.7 Broader Autism Phenotype in parents***

The **Family History Interview** - Subject version (FHI-S; Parr et al., in prep) is a reliable, semi-structured interview used to identify Broader Autism Phenotype behaviours and traits. These are milder ASD related traits sometimes seen in relatives of people with ASD. The FHI asks questions about social, communication and emotional strengths and difficulties in child and adulthood. The FHI-S also includes questions about depression, which will be a measure of mood history.

The primary PACT parent (who took part in the intervention, if in the intervention arm) will be interviewed one-to-one. This will be face-to-face or over the phone. The interview takes between 30 and 60 minutes.

***9.3.8 Service use and Informal care***

The **Child and Adolescent Service Use Schedule** (CA-SUS). Information will be collected from parents at interview using this schedule that was developed and tested in the PACT study. Given

the follow up period we will use a two-stage process successfully used previously, involving the application of the full CA-SUS to the 6-month period prior to interview and a brief version to collect key service data over the whole period since the PACT trial - including services relatively easy to recall over this period such as hospital and residential services and specific therapies for autism treatment. This interview will take place between researcher and parent and takes around 20 minutes.

Parents will also be asked to keep a **Time Diary** over two days (one week day and one weekend day). In this they will be asked to document the care they give to their child across the day and night. This is to measure the amount and type of care families provide for their child.

## 10. ANALYSIS

### *10.1 Sample size*

Participants in the original PACT trial n = 152 were randomised to two groups: (1) Treatment as Usual, and (2) PACT Treatment plus Treatment as Usual. No further random allocation will take place. We aim to trace, contact and recruit at least 80% of the original sample (target n=>120).

### *10.2 Feasibility of follow-up rate*

Previous follow-up studies in autism by the applicants have achieved equal or better retention rates; Charman (Charman et al., 2005) followed-up at age 7 years children who had previously been seen at 2 years and at 3 years of age as part of a preschool early intervention trial. Retention to age 7 years was 90% (26/29). Howlin and Charman (Howlin et al., in prep) followed-up at age 10 years children who had previously been seen at 2 years and at 5 years of age as part of a preschool early intervention study. Retention to age 10 years was 82% (36/44). In Howlin's child-adult study with mean length of follow-up = 37 years32 tracing rate was 91% (82/90) and agreement to participate rate was 67% (60/90).

This study will be adopted by MHRN/CLRN and we will have use of their staff to help with retracing and retention. The families are generally highly motivated to take part in research; since autism is a long-term disorder, families will continue to be concerned and preoccupied with their child's autism.

### *10.3 Power Calculation*

The complexity of the specific context of the study means that the power calculations presented are illustrative. We assume a sample size of 120 (i.e. ignoring the additional power available from the PACT endpoint data for the 32 participants not followed up), two-tailed test and .05 significance criterion. Availability of repeated outcome measurement potentially increases our power. Estimates from PACT and the Early Diagnosis Study (Lord et al., 2006) suggest correlations of ADOS total scores of ~0.7 between baseline, PACT endpoint and follow-up to age 7 years. For estimating a mean difference using ANCOVA with a single pre- and two postrandomisation measures, we have a nominal power of 89% for ES=0.35SD, 96% for ES=0.40SD and 99% for ES=0.5SD (Stata sampsi). In a typical patient cohort we estimate that this last would correspond to a reduction of ~40% of AD and ASD cases being shifted below their corresponding diagnostic threshold and a 7-month improvement in Vineland Adaptive Behaviour Composite. For expressive language and Adaptive Behaviour correlations from baseline to endpoint and follow-up are rather smaller (~0.45) giving corresponding power estimates of 65% for ES=0.35SD, 76% for ES=0.4 and 92% for ES=0.5.For examining the independent effects of a set of prognostic factors for the sample as a whole we will have 78%, 89% and 95% power to detect the effects of a target covariate that explains respectively 3,4 and 5% of the variance in the presence of 4 confounders that explain a further 50% of the variance - plausible given the continuity from baseline measures (Stata, powerreg). For the testing of treatment moderating effects we estimated the power of a treatment group by covariate interaction on a change score (Gpower). Power of 80% requires a difference in standardized slope of 0.21, arguing for the very focused exploration of moderation proposed.

### *10.4 Analysis plan*

Primary analysis of the data will take place within the University of Manchester, King's College London or the University of Newcastle, by Prof Andrew Pickles (Trial Statistician) or Prof Jonathan Green (Chief Investigator). Other team members will have access to the data and undertake analyses as appropriate. Any arrangements for other researchers in the general field to have access to the primary data will be negotiated separately and COREC informed.

Stage 1: Preliminary analyses will approach the follow-up data using the traditional Intention-To-Treat method, covarying for baseline values of measures corresponding to outcomes, and for measures of parental educational qualifications and SES baseline factors, identified previously as unbalanced across treatment groups. The primary outcomes will be: language and communication outcomes (CELF, ROWPVT); family functioning (FLQ) and the ADOS, for which we shall make use of the cross-module standardized severity scores (Gotham et al., 2009). Missing data will be multiply imputed, using only imputations of the baseline variables where the analysis model is maximum likelihood (and thus able to exploit properties of ML for ignorable non-response). Analysis will use appropriate generalised linear models, where possible, using models for repeated measures growth curves fitted to baseline, outcome and follow-up. These will characterise not only an overall effect but also the extent to which later effects are consistent with a magnification or attenuation of the early effects observed in the original trial. Having repeated measures will increase our potential power but, for some analyses, we will need to account for multiple testing (the previous PACT findings essentially forming an interim analysis).

In the case of language outcomes (Hypothesis 1) we will need to account for the floor effects among our baseline measures by using models that allow for censoring (www.gllamm.org). For autism symptoms (Hypothesis 2), growth curve models for ordinal items will be fitted to the ADOS severity scores and factor growth curve models to the ordinal domain scores, in order to detect domain specificity in both development and treatment effects. More standard ordinal item factor models will be fitted to the follow-up data on individual items from the RRB profile, profile of comorbidity diagnoses and for items selected from the Family Life Questionnaire (Hypothesis 3). In addition to assessing the long-term global impact of treatment these factor models allow the assessment of item-specificity of treatment effect in the manner of differential item functioning in more standard item response theory models.

Stage 2: We will use multivariate latent trajectory classification methods (Pickles et al., 2010) applied to the interaction data to characterise treatment responsive and non-responsive parents and dyads and follow through the impact of this early change in the manner of principal stratification (Long et al., 2008; Emsley et al., 2010). We will use this approach to estimate the effect of the treatment-induced change in parental synchrony on language development (Hypothesis 1), and on current child interaction and adaptive functioning (Hypothesis 2).

Stage 3: We will extend analyses from stage 1 to examine interactions reflecting the potential impact of moderator factors particularly highlighting parental BAP.

## 11. DATA MANAGEMENT

### *11.1 Data management*

The custodian will be Professor Jonathan Green, Chief Investigator of the study. The data will be accessed only by current and future members of the PACT research team. All data in the study will be anonymised. A central master data-file will be held centrally by the study team at the Jean McFarlane Building, University of Manchester, Oxford Road, Manchester M13 9PL. This file will contain the key linking anonymised participant number to personal details. This file will be backed up on the secure university server. All data will be double entered into a web based secure data entry system. The data will be held for 10 years.

### *11.2 Data protection and confidentiality*

All research staff will be given training in data protection and confidentiality procedures. Data protection guidelines will be followed at all times.

Datasets will not contain person-identifiable information (names, addresses). Participant data will be identified via an identification number. The database linking names and identification numbers will be stored separately to the main datasets and will be encrypted and held securely at each site. No names or personal identifiable information will appear within written output. The results will consist of group level analyses only; groups will contain relatively large numbers of participants. No case studies or histories will be published.

Electronic personal data files (e.g. database of contact details) will be encrypted and stored on an external hard-drive at each site. The hard-drive will be kept in a locked cabinet within a secure university or NHS building and will not be removed from the premises. Hard copies of electronic personal data (ie DVDs of video recordings of assessments) will be kept in a locked cabinet within a secure university or NHS building. Electronic video/audio material will sometimes need to be shared amongst research staff (e.g. to check reliability on coding measures). In these circumstances, the files be encrypted and will be transported between sites as securely as possible. Paper copies of personal information (e.g. consent form, contact details, summary reports) will be kept in a locked cabinet within a secure university or NHS building and will not be removed from the premises. The exception is when material is being posted to families or key professionals (e.g. summary report). Person-identifiable information will be stored securely, as detailed above. Paper documentation and video material will be transported as directly as possible from its source (home or clinic visit) to be stored securely on university or NHS premises.

Information about participants will not be shared with individuals or organisations outside of the research team without parental consent. The child's GP will be informed of the family's participation in the study and consent for this will be obtained on the consent form. The child's school will also be contacted and informed of the family's participation in the study and consent for this will be obtained on the consent form. The family will receive a written report of the assessments and they will be free to share this with whomever they choose.

The only exception to the rule of confidentiality is if there are risk issues. It is possible that the research team may become aware of potential risk to the child (or parent) during the course of the research, either through disclosure from the child or parent, or through witnessing evidence of neglect or abuse. Parents will be informed through the Participant Information Sheet and through discussion with the researcher at the time of consent that all information will be kept confidential except if the researcher has reason to believe that an individual is at risk. In the event of any safeguarding concerns, the researcher will discuss with the parent who could be informed. Research staff will discuss any risk issues with their supervisor (who will be a senior clinical academic at each site) at the next available opportunity. This will be immediately over the telephone in the event of urgent concerns. A team decision would then be made and normal clinical protocols followed (these provide for families to be informed about any referral to – for instance – statutory safeguarding teams prior to this happening, except in the most extreme circumstances of risk to child).

## 12. STUDY MONITORING

### *12.1 Steering Committee*

The steering committee will include: Professor Eric Taylor (Institute of Psychiatry), Mr Richard Mills (Collaborator, National Autistic Society), representatives from the local National Autistic Society, Lindsay Stairs (Parent Representative), Bridget Gilchrist (Parent Representative), Professor Jonathan Green (CI), Professor Andrew Pickles (Statistician), and other team members as appropriate.

The steering committee will be consulted on the final design of the follow-up, techniques for ascertainment and the focus for measurement. We will have a particular focus on ascertaining families' experience and quality of life using our new measure, the FLQ, developed at MRC request within the PACT trial.

### *12.2 Project management and data monitoring group*

The project management group will be chaired by Professor Green and consist of the Principal Investigators and senior researchers on the study and other invited members as necessary. It will meet quarterly.

### *12.3 Monitoring and indemnity*

The study will be subject to the audit and monitoring regime of the University of Manchester. The University of Manchester will arrange insurance for research involving human subjects that provides cover for legal liabilities arising from its actions or those of its staff or supervised students, subject to policy terms and conditions.

### *12.4 Conflicts of interest*

None of the participating PACT families or children are seen clinically by members of the research team.

## 13. DISSEMINATION

The results of the research will be targeted for publication in peer-reviewed journals of general and special interest. There will also be a general dissemination programme for families including participants co-ordinated through our collaborators in the National Autistic Society. For the families and local referring clinicians involved in the study, we send 6-monthly newsletters updating them on the progress of the study, when the findings will be published and other locally and nationally relevant autism 'news'. A written summary of the study results will be sent to all participants in the form of a newsletter at the end of the study. We will publish our findings on the study website (http://www.bbmh.manchester.ac.uk/pact/). We will also disseminate results through local clinical teams (by whom the original PACT referrals were made). At the end of the original PACT trial all 3 collaborating sites held local meetings/conference for parents, professionals, service managers and commissioners to disseminate the trial findings. The same range of dissemination events will be arranged via local services and service users for the end of this follow-up study.

A number of the investigators are active nationally and internationally in communication with parents and professionals more widely (for example, McConachie and Le Couteur run a Database of children with ASD in the North East (Daslne); this provides an information website, and sends newsletters twice yearly to 1500 families and professionals). Many of the team present regularly at Special Interest Groups for clinical professionals, for example, the London Special Interest Group for speech and language therapists has over 100 members working across the whole of the South East. At key points at the beginning of the study and when its findings are published we will use these mechanisms to communicate with relevant parent and practitioner audiences.

## 14. FINANCE

MRC research funding - £840,251

## 15. ETHICAL APPROVAL AND CONSIDERATIONS

### *15.1 Ethical Approval Information*

REC Reference number: 13/NW/0144

### *15.2 Procedures for minimising negative consequences*

In order to minimise disruption and inconvenience to children and parents we will offer a range of options for home/ clinic visits including early evening, weekends, school holidays or during the day. If it is most convenient for children to be assessed during the school day, a letter on headed paper will be sent to the family to justify the child's absence to the school. The balance between the number and length of appointments will be discussed with the parents at the outset to achieve the best balance for them as a family. Researchers will be sensitive to several issues when arranging visits, including family or work commitments, childcare, and time burden on the parent. Parents will be given the option to complete the assessments at a later date if it is felt that they are struggling during the course of the visit.

During interviews researchers will be aware of the fact that they cover topics that parents may find difficult or upsetting. During interviews, researchers will present questions in a sensitive way and offer the parent the chance to take a break or halt the interview if the parent becomes upset. At the end of an assessment researchers will offer the opportunity to debrief if appropriate. Parents who complete the DAWBA online will be provided with a phone number to call the local research team if they have any concerns or questions when completing the DAWBA. The researcher will then support and reassure parents who have concerns or become upset and will seek advice from their supervisor if any serious concerns are raised. Participants will be informed at the start of the Family History Interview (FHI) that researchers will be happy to talk to them about any concerns that arise as a result of the interview, and if they are still concerned, the researcher will signpost to appropriate sources of support, such as consulting their GP. We will also have a named clinician, a member of the PACT team who is familiar with the FHI and Broader Autism Phenotype, that the parent can speak to afterwards, should they wish to speak to someone about their concerns.

The clinic visit will be made as enjoyable and stress-free for children as possible. Parents will be asked to go through the information sheet with the child prior to the visit. The clinic room will be relatively distraction-free. The parents will be able to stay in the room with the child if this is required to avoid distress. Regular breaks will be offered to the child, in discussion with the parent, to accommodate their needs (toileting, snacks, need to move around or have time-out). If it becomes clear at any point that the child has reached their tolerance level, again in discussion with parents, the session will be ended and continued at a later date.

Researchers will receive lone working training prior to commencement of visits and will follow these procedures when visiting homes or other settings. If for any reason it is felt that the risk is higher than acceptable (e.g. from prior knowledge of family or local area; from information gleaned during previous contacts), a second researcher will accompany the first on the visit or alternative plans will be made. All researchers will have a work mobile phone and will be contactable throughout home visits. All staff working on this study will hold a valid Criminal Record Bureau check. Parents will be present or within the immediate vicinity whilst assessments with children are completed in order to protect children and researchers.

## 16. REFERENCES

1) Green J. Charman, T. McConachie, H. et al (2010). Parent-Mediated Communication-Focused Treatment for preschool children with Autism (PACT); a randomised controlled trial. *The Lancet*, 375(9732), 2152-2160.

**PACT 7-11 SUMMARY OF PROTOCOL AMENDMENTS**

The following changes have been made to the protocol from v.1 to v3.

| ***Version Number*** | ***Date*** | ***Section*** | ***Amendment*** |
|---|---|---|---|
| v.2 | 05.09.13 | 1. General Information, 12. Study Monitoring and 13. Dissemination | These sections have been updated to reflect the following changes: new website address; addition of new Research Associate; change to Trial Steering Committee members. |
| | | 7. Timeline | The timeline has been changed to allow more time for start-up tasks and staff training (from April 2013 to June 2013). |
| | | 8.3. Assessment Procedures and 9.3 Measures | The SF36v.2 Health Survey has been replaced by the Parent Wellbeing Questionnaire. These sections have been changed to reflect this change. Details of the Parent wellbeing Questionnaire have been added. |
| | | 8.3. Assessment Procedures and 9.3 Measures | The Manchester Playground Observation Measure (MIPO) has been added to the protocol and new information about this measure has been added in these sections. |
| v. 3 | 22.11.13 | 8.3 Assessment Procedures and 9.3 Description of Measures | The Autism Diagnostic Interview-Revised (ADI-R) is no longer part of the assessment battery and has been replaced by the Social Communication Questionnaire (SCQ) and Repetitive Behaviour Questionnaire (RBQ). Therefore the ADI-R information has been deleted and details of the SCQ and RBQ have been added in these sections. |

# PACT 7-11 ANALYSIS PLAN FOR THE PRIMARY FOLLOW-UP PAPER

**Authors:** Pickles A, Le Couteur A, Leadbitter K, Salomone E, Tobin H, Cole-Fletcher R, Gammer I, Lowry J, Vamvakas G, Byford S, Aldred C, Slonims V, McConachie H, Howlin P, Parr J, Charman T, Green J.

**Agreed 21/1/15 prior to inspection of follow-up data by treatment group**

**Appendix 1: Major post application revisions incorporated in this version**

**Appendix 2: Subsequent revisions occurring post data inspection**

**Document Revised for publication 29/1/16**

## STUDY DESIGN AND TRIAL ENDPOINT ANALYSIS

As described in Green et al. (2010).

## FOLLOW-UP ANALYSIS

### Overall strategy

Intention-to-Treat analysis of original trial/randomization design.

Primary outcomes: ADOS, DCMA, Language composite
Secondary outcomes: VABS, RBQ, FLQ, DAWBA, SDQ, SCQ

Analysis goal: to use all the data to obtain good estimates of outcome where possible using growth curve approaches to yield a group difference at the median follow-up age (elaborated in PART A below) or as a simple follow-up difference or latent regression (elaborated in PART B below). No imputation of baseline data required.

### Description

Follow-up rates to be described and balance in baseline covariates among those followed up to be described. CONSORT type diagram in on-line supplement.

Table 1 with Baseline and Follow-up mean/proportion/median and SD/range by treatment group for gender, age-at-recruitment, age-at-follow-up, numbers by centre.

Table 2 Follow-up mean/proportion/median and SD/range by treatment group for primary and secondary measures.

Treatment differences to be summarized in a tree-plot like effect size diagram.

**Part A**

**Growth Curve Analysis model**: For **repeated measures** (i.e. those already measured in PACT) treatment group comparisons at the mean age at follow-up based on a piecewise growth curve model with separate correlated pieces over PACT and PACT7-11. Baseline variables for which groups are imbalanced will be included as fixed-effect covariates with interactions of group and time to allow different effects during PACT and PACT7-11. Group differences will be estimated at the median follow-up age.

*Primary Outcomes*

**1. ADOS-2** overall Comparative Severity Score (CSS)[2]. Then report Social Affect and Restricted and Repetitive Behavior Comparative Severity Scores[3] descriptively. The numbers completing each ADOS Module will be reported.

*Analysis specifics*: The follow-up CSS total scores will be analysed together with baseline and trial endpoint CSS scores, as a set of correlated ordinal scales with different thresholds with group main-effect and time by group interaction, with random intercept and random piecewise slopes, covarying for imbalanced baseline variables. Estimated by maximum likelihood missing data will be assumed Missing at Random.

**2. DCMA[4] PCI from PLAY only**

Primary: Child initiations as per 2010 paper (proportion of child codes)
Secondary: Parent synchronicity as per 2010 paper (proportion of adult codes)
Secondary: Child conversation as a frequency
Secondary: Mutual shared attention.

*Analysis Specifics*: Coded reliably only in the 8min PLAY session, synchronicity and initiations at baseline, trial endpoint and follow-up will be analysed by using a mixed effects model with random piecewise time slopes and group by time interactions covarying for imbalanced baseline variables. Stata lincom will be used to construct a single effect estimate at the median age of follow-up. Mutual shared attention and conversational frequency will be presented descriptively.

*Secondary Outcomes*

**3. Parent and Teacher VABS standard score**

Parent Vineland Adaptive Behavior Composite[5] at baseline, trial endpoint and follow-up will be analysed by using a mixed effects model with piecewise time slopes and group by time interactions for imbalanced baseline variables. Stata lincom will be used to construct a single effect estimate at the median age of follow-up.

Teacher Vineland Adaptive Behavior Composite at follow-up will be analysed by regression covarying for imbalanced baseline variables.

**4. FLQ total score**

The Family Life Questionnaire total score will be analyzed in the same manner as Parent VABS, but this measure lacked a published reference.

**Part B**

For outcomes that are novel to PACT7-11 (i.e. lacking baseline equivalents) group comparisons based on regression/SEM latent regression of PACT7-11 score on imbalanced covariates, age at follow-up and any baseline measure similar to outcome.

*Primary Outcomes*

**5 Language factor composite**

Descriptive statistics by group for the CELF[6] subtests (including number not administered), and 2 One-Word tests[7] will be given. Using Mplus, a preliminary factor analysis will be described and a jointly estimated factor model with treatment/covariates effects estimated.

*Secondary Outcomes*

**6. Social Communication Questionnaire and Repetitive Behavior Questionnaire totals**

Regression of total scores[8,9] on group covarying for any imbalanced covariates.

**7. DAWBA – SDQ**

1. *DAWBA bands*

For each disorder rated the DAWBA[10] produces bands indicating the percentage of children with these scores who (from the ONS datasets) would meet DSM/ICD criteria for that particular disorder:
Level 0: <0.1% of children in this band have the disorder
Level 1: ~0.5% of children in this band have the disorder
Level 2: ~3% of children in this band have the disorder
Level 3: ~15% of children in this band have the disorder
Level 4: ~50% of children in this band have the disorder
Level 5: >70% of children in this band have the disorder

- ICD-10 disorders will be grouped into four categories (any anxiety or OCD; conduct or oppositional; hyperkinesis; depression). In multiple disorder categories the disorder with the highest Likelihood level will be used.
- Data will be presented in two Venn diagrams, for PACT and TAU, using the cutpoint of >50% for disorder present. Group differences in rates assessed using ordinal logistic regression.

2. *Strength and Difficulty Questionnaire scores*

SDQ[11] Peer problems and prosocial scores to be analysed by ordinal regression for group difference, covarying for imbalance baseline covariates.

**References**

(1) Green J, Charman T, McConachie H, Aldred C, Slonims V, Howlin P, Le Couteur A, Leadbitter K, Hudry K, Byford S, Barrett B, Temple K, Macdonald W, Pickles A; PACT Consortium. (2010) Parent-mediated communication-focused treatment in children with autism (PACT): a randomised controlled trial. Lancet 19, 2152-60. doi: 10.1016/S0140-6736(10)60587-9.
(2) Gotham K, Pickles A, Lord C. Standardizing ADOS Scores for a Measure of Severity in Autism Spectrum Disorders. J Autism Dev Disorder 2009;39:693-705.
(3) Hus V, Gotham K, Lord C (2014) Standardizing ADOS domain scores: separating severity of social affect and restricted and repetitive behaviors. J Autism Dev Disord. 2014 Oct;44(10):2400-12. doi: 10.1007/s10803-012-1719-1.
(4) Aldred C, Green J, Emsley R, McConachie M. Brief report: mediation of treatment effect in a communication intervention for pre-school children with autism. J Autism Dev Disorder 2011;42:447-54
(5) Sparrow S, Cicchetti D, Balla D. Vineland adaptive behavior scales: (Vineland II), survey interview form/caregiver rating form. Livonia, MN: Pearson Assessments 2005.
(6) Semel E, Wiig E, Secord W. Clinical evaluation of language fundamentals. 7th ed, Australian standardised edition (CELF-4 Australian). Sydney, Australia: Pearson Inc, 2006.
(7) Receptive and Expressive One-Word Picture Vocabulary Tests, Fourth Edition (ROWPVT-4, EOWPVT-4). Brownell. San Antonio TX: Pearson Education, 2010
(8) Rutter M, Bailey A, Lord C. Social Communication Questionnaire. 2003. Los Angeles, CA: Western Psychological Services.

(9) Honey E, McConachie H, Turner M, Rodgers J. Validation of the repetitive behaviour questionnaire for use with children with autism spectrum disorder. Res Autism Sepctr Disord 2012;6:355-64
(10) Goodman A, Heiervang E, Collishaw S, Goodman R. (2011). The 'DAWBA bands' as an ordered-categorical measure of child mental health: description and validation in British and Norwegian samples. *Soc Psychiatry Psychiatr Epidemiol*. 2011 Jun;46(6):521-32. doi: 10.1007/s00127-010-0219-x
(11) Goodman R. The Strengths and Difficulties Questionnaire: A Research Note. J Child Psychol Psychiatry 1997;38:581-6.

## APPENDIX 1: Major post application revisions incorporated in this version

**Outcomes:**
The principal outcome, the ADOS CSS, remains unchanged.
The language outcome remains unchanged.
The FLQ is demoted from primary to secondary in the light of concern about no available publication for the measure.

Measures from the DCMA measuring mother-child interaction are promoted in the light of other work on the mechanism analysis of the PACT trial, that highlighted these measures in understanding treatment mechanism (Pickles et al 2014). Of the available codes, Child Initiations was considered the best proximal outcome, given the results of this mechanism analysis. Conversational turns and mutual shared attention were included as child secondary outcomes. Parent Synchrony was included as a secondary informative as to treatment mechanism. Measures to be constructed as in the original trial.

With the FLQ demoted, but an ongoing concern to represent the parent perspective, potential generalization of effects into the school domain and growing awareness of comorbidities the parent and teacher Vineland ABC, the parent reported SCQ and RBQ, and SDQ and DAWBA are added as additional secondary outcomes.

## APPENDIX 2: Subsequent revisions occurring post data inspection

During analysis:

(1) Two secondary outcomes were dropped: i) Mutual Shared Attention from the DCMA interaction coding that had been suggested for descriptive reporting but on later testing in independent middle childhood samples had been found to be unreliable and age-inappropriate; ii) family life score from the FLQ questionnaire for which published validation had not yet become available;

(2) For the longitudinal models the Area-Under-the-Curve estimates were added as a means of summarizing treatment effect over the course of the study and multiple imputation and GEE/robust standard error estimation was used as an additional check as the robustness of the findings to model misspecification and missing data;

(3) A proposed Venn-diagram spanning five categories of co-occurring disorders from the DAWBA was dropped as being too disaggregated to be interpretable;

(4) Bootstrap standard errors were used for the confidence intervals of all effect estimates as their properties were likely to be superior to those based on standard asymptotics for many of the statistics presented;

(5) The inclusion of dummy variables for centre effects.
